# Notch signaling in liver diseases: mechanistic insights and therapeutic implications

**DOI:** 10.3389/fcell.2026.1770031

**Published:** 2026-03-09

**Authors:** Qingmiao Shi, Na Lou, Di Huang, Leiya Fu

**Affiliations:** 1 Department of Infectious Diseases, The First Affiliated Hospital, College of Clinical Medicine, Henan University of Science and Technology, Luoyang, China; 2 Department of Infectious Diseases, The First Affiliated Hospital of Zhengzhou University, Zhengzhou, China; 3 Department of Child Health Care, The Third Affiliated Hospital of Zhengzhou University, Zhengzhou, China

**Keywords:** liver cancer, liver diseases, liver fibrosis, non-alcoholic fatty liver disease, notch signaling, treatment strategies

## Abstract

The Notch signaling pathway represents an evolutionarily conserved mechanism of intercellular communication that plays critical roles in organ development and tissue homeostasis. However, its functions in liver physiology and pathology demonstrate remarkable context-dependent characteristics, with dysregulated signaling contributing to various liver disorders. This review systematically summarizes the complex roles of Notch signaling in liver health and disease. It comprehensively examines the pathway’s essential functions in biliary development, hepatic regeneration, and metabolic homeostasis, while providing a detailed analysis of its pathogenic mechanisms in conditions including Alagille syndrome, drug-induced liver injury, non-alcoholic fatty liver disease, liver fibrosis, hepatocellular carcinoma, and intrahepatic cholangiocarcinoma. Our review particularly emphasizes the dual function of Notch signaling in hepatobiliary malignancies, where it can exert either oncogenic or tumor-suppressive effects depending on specific cellular contexts, molecular interactions, and microenvironmental cues. Furthermore, we highlight the evolution of therapeutic strategies from broad-spectrum γ-secretase inhibitors to more precise approaches involving ligand-specific antibodies, transcriptional complex blockers, and pathway agonists, while addressing persistent challenges in clinical translation including on-target toxicities, compensatory resistance mechanisms, and context-dependent responses. Looking forward, this review outlines promising research directions featuring biomarker-guided patient stratification, rational combination therapies with immune checkpoint inhibitors, and spatiotemporally precise regulatory strategies. By integrating foundational knowledge with recent advances, this work provides valuable insights for understanding Notch signaling’s complex roles in liver pathophysiology and for developing novel therapeutic interventions.

## 1 Introduction

Liver disease represents a major global health concern, leading to approximately two million annual deaths worldwide (Xiao et al., 2025). The burden of liver disease is shifting rapidly from traditional viral hepatitis to alcohol-related liver disease (ALD) and non-alcoholic fatty liver disease (NAFLD) (Devarbhavi et al., 2023). NAFLD, as the most common chronic liver disease worldwide, can progress to more severe forms, including non-alcoholic steatohepatitis (NASH), liver cirrhosis, and even hepatocellular carcinoma (HCC) (He et al., 2025; Xu and Wang, 2021). Although substantial progress has been made in understanding and treating liver diseases globally, numerous challenges still persist in the face of an increasingly complex disease spectrum and medical burden. Therefore, there is an urgent necessity for international collaboration to improve prevention, diagnosis, and treatment strategies (Wang et al., 2014).

The Notch signaling pathway is a highly conserved intercellular communication mechanism that regulates fundamental cellular processes including proliferation, differentiation, and apoptosis (Shi et al., 2024a). The pathway comprises four receptors (Notch1-4) and five ligands (JAG1/2, DLL1/3/4). Activation of the canonical Notch pathway occurs when the γ-secretase enzyme complex cleaves the Notch receptor, releasing the Notch intracellular domain (NICD) (Chen et al., 2025; Martinez Lyons and Boulter, 2023; Sachan et al., 2024; Sprinzak and Blacklow, 2021). NICD subsequently translocates to the nucleus and forms a transcriptional activation complex with CSL/RBP-Jκ to initiate expression of target genes, including Hes and Hey families. In addition to its canonical signaling route, the Notch pathway can also function through non-canonical pathways that function independently of CSL/RBP-Jκ (Zhang et al., 2024a). These non-canonical signaling mechanisms often interact with other critical pathways, further increasing the complexity of Notch pathway. Dysregulation of Notch signaling contributes to epithelial-mesenchymal transition (EMT), angiogenesis, cancer stem cell maintenance, metabolic reprogramming, and chemotherapy resistance across various cancers (Fu et al., 2025; Mu et al., 2024). This pathway exhibits remarkable context-dependent functions, serving dual roles as either an oncogene or tumor suppressor across different tissues and malignancies (Li et al., 2023a; Suarez Rodriguez et al., 2023). Notch signaling depends on specific receptor-ligand interactions, and its functional impact varies significantly across different cell types (Kopan and Ilagan, 2009). Furthermore, the signaling outcome is further regulated by the cellular microenvironment, including factors such as the extracellular matrix, neighboring cells, and cytokines. Importantly, the function of Notch signaling also displays a strong temporal dependency, changing across developmental stages and physiological processes. In the liver, this context-dependent functionality is particularly evident (Meurette and Mehlen, 2018). For example, it suppresses tumor development in squamous cell carcinomas but promotes cancer progression in certain hematological malignancies like T-cell acute lymphoblastic leukemia (Shi et al., 2024b). This contextual duality makes Notch signaling both a challenging therapeutic target and a promising biomarker for predicting cancer outcomes and assessing treatment responses.

The Notch signaling pathway plays a significant role in organ development across diverse species and is recognized as essential for mammalian liver development, regeneration, and disease (Adams and Jafar-Nejad, 2019). Its aberrant activation disrupts energy homeostasis, thereby inducing metabolic disorders and promoting the progression of conditions such as obesity, NAFLD, and NASH (Xi et al., 2025). Additionally, studies indicate that dysregulated Notch signaling is directly involved in the progression of various liver diseases, including hepatoblastoma, cholangiocarcinoma, and HCC (Valizadeh et al., 2021). In HCC specifically, Notch signaling plays a key role in regulating the tumor microenvironment (TME), tumorigenesis, progression, angiogenesis, invasion, and metastasis (Huang et al., 2019). Overactivation of Notch signaling is closely associated with reduced survival rates and poorer clinical outcomes in HCC patients. The underlying pathological mechanisms are complex and are precisely controlled by upstream regulatory molecules. The long non-coding RNA NEAT1 has been identified as a significant modulator to liver diseases, functioning through the regulation of key signaling pathways including Notch, Wnt, and nuclear factor kappa-B (NF-κB), either directly or via the sponging of regulatory miRNAs (Saleh et al., 2024). Furthermore, cellular reprogramming during acute and chronic liver injury, which governs the lineage switch between hepatocytes and cholangiocytes, is also closely related to Notch-regulated tissue regeneration (Aoshima and Tanimizu, 2025).

This review aims to systematically summarize the role of the Notch signaling pathway in liver physiology and various liver diseases. It highlights recent experimental and clinical findings that elucidate the involvement of the Notch signaling pathway in key pathological processes, including EMT, cancer stem cell characteristics, and immune microenvironment. Furthermore, the review assesses the current landscape and challenges of therapeutic strategies targeting Notch signaling and explores their therapeutic intervention. By integrating these insights, this review aims to offer novel perspectives and theoretical frameworks that may enhance future strategies for the diagnosis, prevention, and treatment of liver diseases.

## 2 Notch signaling pathway in liver physiology

The Notch signaling pathway, an evolutionarily conserved mechanism of cell-cell communication, plays a central regulatory role in liver development, regeneration, and the maintenance of homeostasis. During embryonic liver development, the Notch signaling pathway orchestrates the remodeling of the ductal plate and the formation of intrahepatic bile ducts (Kodama et al., 2004; Martinez Lyons and Boulter, 2021; Sparks et al., 2010). Notably, organoid models confirm the high plasticity of the Notch pathway in biliary development and disease modeling (Koike et al., 2022; Peng et al., 2025). In the repair of adult biliary injury, activation of the Notch pathway mediated by ligands Jagged1 and DLL4 promotes the ductular reaction and the differentiation of hepatic progenitor cells (HPCs) into biliary epithelial cells (BECs) (Martinez Lyons and Boulter, 2023). Hepatic stellate cells (HSCs), as key components of the HPC niche, activate Notch signaling by expressing Jagged1, thereby precisely regulating the differentiation fate of HPCs (Kitade et al., 2019). Aberrant activation of Notch signaling disrupts energy homeostasis and participates in the progression from NAFLD to liver fibrosis and HCC (Valizadeh et al., 2021). Furthermore, oxidative stress has been shown to activate the Notch pathway, thereby regulating the proliferation and metastasis of HCC cells (Li et al., 2023b). In pathological states such as acute-on-chronic liver failure, the DLL4-Notch signal participates in the liver injury response by regulating immunometabolism and biliary regeneration (Zhou et al., 2023b). Significantly, during liver regeneration following injury, the Notch pathway interacts with Hippo/YAP and Wnt/β-catenin signals, thereby responding to mechanical stimuli like portal vein shear stress. It is activated via the Piezo1 channel to regulate the balance between hepatocyte hyperplasia and hypertrophy (Valizadeh et al., 2019). Moreover, in the fibrotic liver microenvironment, the TNFα/NF-κB axis induces hepatocytes to express DLL4, thereby promoting extramedullary T cell development (Gong et al., 2019). In summary, through spatiotemporally controlled activation patterns, the Notch pathway integrates developmental, metabolic, immune, and mechanical signals, playing a core regulatory role in liver physiology and pathological repair.

## 3 Notch signaling pathway in non-tumor diseases of the liver

The Notch signaling pathway is a pivotal regulator in both neoplastic and non-neoplastic liver diseases, demonstrating remarkable functional plasticity that influences pathogenesis and progression (Schwabe et al., 2020). Identifying these shared mechanisms contributes to a clearer understanding of liver diseases and may provide practical insights for therapeutic targeting. The following sections examine the regulation of Notch signaling in non-tumor diseases of the liver, revealing both unique pathological mechanisms and common therapeutic targets (Table 1).

**TABLE 1:** Mechanism of the Notch pathway in non-tumor diseases of the liver.

Disease	Notch component	Involved molecules	Mechanism	Reference
ALGS	JAG1	Sox9b/SOX9	JAG1 deficiency→ Notch signaling attenuation→ Sox9 expression suppression→ biliary regeneration failure	Zhao et al. (2022)
ALGS	JAG1, Notch1, Hes1	Sox9	ASO upregulates JAG1→ activates Notch signaling pathway→ improves bile duct development	Zhang et al. (2025a)
AILI	NICD	PTEN, NRF2, STING	Macrophage PTEN deletion→ NICD/NRF2 nuclear interaction→ STING pathway inhibition→ reduced inflammation/necroptosis	Yang et al. (2023)
AILI	Notch2	MSCs, COX2, PGE2, AMPK, Sirtuin 1, XBP1s, NLRP3	JAG1-activated MSCs→ Notch2/COX2/PGE2→ macrophage AMPK/SIRT1 activation→ XBP1s deacetylation→ NLRP3 inhibition→ alleviates liver injury	Yu et al. (2022)
AILI	Notch1, JAG1, Hes1	Matrine	Matrine inhibits Notch signaling pathway→ ↓Hes1→ promotes differentiation of hepatic oval cells into hepatocytes→ alleviates liver injury	Shi et al. (2020)
AILI	Notch1, Hes1	PCPCF	PCPCF inhibits Notch signaling pathway→ ↓Hes-1→ repairs intestinal mucosal barrier→ improves gut-liver axis→ alleviates Aflatoxin B1-induced liver damage	Tao et al. (2023)
HIRI	Notch	EMCN, LFA-1, TNF-α	Notch activation→ EMCN downregulation → enhanced neutrophil adhesion/migration via LFA-1→ aggravated inflammation→ exacerbated liver injury	Zhang et al. (2020)
HIRI	Notch1, NICD	β-catenin, TRAF6, TAK1, NF-κB, RIPK3, MLKL	Activates Macrophage Notch1 → NICD release and nuclear translocation → activates β-catenin→ TRAF6/TAK1 signaling → reduces inflammation→ Attenuates liver injury	Xu et al. (2022)
HIRI	Notch1, JAG1, HSF1	Snail, TXNIP, NLRP3, Caspase-1, thioredoxin, ASK1, ROS	JAG1/Notch1/HSF1/Snail aix→ inhibits TXNIP/NLRP3/caspase-1 inflammasome activation→ Hepatocyte apoptosis ↓	Jin et al. (2020)
HIRI	Notch1	LRP1, C/EBPβ, SASP	RRPGLY inhibits LRP1-Notch1-C/EBPβ →ameliorates HIRI	Zhang et al. (2024b)
HIRI	Notch1, JAG1	SOX9	MSCs-EXO→ JAG1/Notch1/SOX9 signaling activation→ reduces oxidative stress and apoptosis	Yasen et al. (2023)
NAFLD	HES5	SIRT1, LIGHT/TNFSF14, LTβR	Pro-NAFLD stimuli→ ↓ HES5 expression →↑ LIGHT transcription → LIGHT binds to LTβR on hepatocytes → ↑ Hepatocyte apoptosis	Miao et al. (2021)
NAFLD	Notch1	SREBP-1c	Dietary lingonberry supplementation → Inhibits hepatic Notch1 signaling →promotes fatty acid oxidation	Madduma Hewage et al. (2022)
NAFLD	Notch1, Hes1	PRDX6, ROS	PRDX6 supplementation → ↓ ROS → maintains mitochondrial function → ↓ Notch1 signaling → ↓ lipogenesis/↑ fatty acid oxidation → improved NAFLD	Lee et al. (2019)
NAFLD	RBP-J, HES1, HEY1	IL-1β, TNF-α	Myeloid-specific RBP-J deletion or exosome-delivered RBP-J Decoy ODNs → inhibits Notch Signaling → ↓ IL-1β/TNF-α secretion → ameliorates NAFLD.	Ding et al. (2023)
Liver fibrosis	JAG1	TLR4, NF-κB,ASO	NASH Diet →Hepatocyte TLR4 → triggers NF-κB signaling pathway→ transcriptional upregulation of JAG1 → Notch Signaling →liver fibrosis	Yu et al. (2021)
Liver fibrosis	NICD, RBP-J	Sox9, Opn	NASH → activation of hepatocyte Notch → increase in Sox9 → increase in Opn secretion → activation of hepatic stellate cells → hepatic fibrosis	Zhu et al. (2018)
Liver fibrosis	NICD, RBP-J	MCP-1	NASH → activates hepatocyte Notch signaling → directly upregulates MCP-1 (CCL2) transcription→ promotes HSC activation → liver fibrosis	Kang et al. (2023)
Liver fibrosis	HES1, HEYL	CHCHD2, OPN/SPP1, YAP/TAZ-TEAD1	NASH stimuli→ upregulates CHCHD2 expression via YAP/TAZ-TEAD1→ Notch signaling→ Increases OPN secretion → activates HSCs → liver fibrosis	Li et al. (2022)
Liver fibrosis	N1ICD, HES1	POFUT1, Fibrinogen, STAT3	POFUT1 deficiency → activates Notch signaling → upregulates STAT3 phosphorylation → increases fibrinogen synthesis → activates HSCs via integrin αvβ3/5 → liver fibrosis	He et al. (2024)
Liver fibrosis	Notch3, Hes1	YAP, αSMA, COL1A1	TKF → inhibits YAP → downregulates Notch3-Hes1 signaling → suppresses HSC activation and fibrogenic gene expression → ameliorates fibrosis	Yang et al. (2022)
Liver fibrosis	Hes1, RBP-J	αSMA, COL1A1	MF-specific RBPj KO → inhibits HSC activation to MFs and ↑MMPs/↓TIMP1 → ↓ECM deposition → attenuates fibrosis.​	Yue et al. (2021)
Liver fibrosis	RBP-J	IL-1β, IL-6, TNF-α, PDGF-B, TGF-β, Col1a1, αSMA	Activation of RBP-J→ regulates macrophage activation→ modulates the hepatic immune microenvironment → indirectly influences fibrosis progression	He et al. (2022)

### 3.1 Alagille syndrome (ALGS)

ALGS is an autosomal dominant, multisystem disorder primarily caused by pathogenic variants in genes associated with the Notch signaling pathway, most commonly *JAG1* and, less frequently *NOTCH2* (Kohut et al., 2021). The disease is characterized by a broad and highly heterogeneous clinical spectrum involving multiple organ systems, including the liver, heart, vasculature, skeleton, and kidneys, as well as distinctive craniofacial and ocular abnormalities. A hallmark hepatic pathology is intrahepatic bile duct paucity, which leads to chronic cholestasis (Turnpenny and Ellard, 2012). Extensive studies demonstrate that the intact JAG/Notch signaling axis plays a central role in the development of intrahepatic bile ducts. Genetic disruption of this signaling axis is recognized as the primary molecular cause of the structural and functional abnormalities observed in ALGS.

A previous study identified six novel pathogenic mutations in Chinese pediatric patients with ALGS, emphasizing the importance of genetic testing in early diagnosis (Chen et al., 2024a). Furthermore, Feng et al. reported two Chinese cases of ALGS carrying novel *JAG1* variants, further highlighting the essential role of the Notch signaling pathway in the development of the biliary system, heart, and skeleton (Feng et al., 2024). Importantly, Ferrandino and colleagues revealed that missense and null mutations in rare *NOTCH2* variants disrupted Notch receptor function, thereby contributing to ALGS pathogenesis. Their findings further indicated that phenotypic variability may be influenced by both mutation type and the presence of modifier genes (Ferrandino et al., 2024).

Sox9, a key transcription factor, serves as a direct downstream effector of the Notch signaling pathway. In ALGS, mutations in *JAG1* or *NOTCH2* impair Notch signaling, resulting in insufficient activation of Sox9 expression. Consequently, disruption of the JAG/Notch/Sox9 axis compromises the branching and segregation of regenerating cholangiocytes, ultimately leading to the characteristic intrahepatic bile duct paucity and cholestasis (Zhao et al., 2022). Furthermore, YAP1, a transcriptional co-activator positioned downstream of the Notch pathway, plays a crucial role in the polarization and maturation of the bile ducts. Loss of YAP1 leads to incomplete ductal development despite preserved initial cell fate specialization mediated by Notch signaling (Molina et al., 2021). Given that monoallelic pathogenic variants in *JAG1* constitute the primary genetic cause of ALGS, therapeutic strategies aimed at directly increasing JAG1 protein levels represent a potential etiology-directed treatment strategy. Zhang et al. demonstrated that targeting *JAG1* mRNA with antisense oligonucleotides (ASOs) effectively enhances JAG1 protein expression and Notch signaling activity, which improves bile duct development and liver function in an ALGS mouse model (Zhang et al., 2025a). In summary, these findings underscore that ALGS is fundamentally a disorder resulting from impaired Notch signaling. Emerging therapeutic strategies aimed at restoring this pathway, such as ASO-mediated enhancement of JAG1 expression, hold significant promise for addressing the underlying pathophysiology of the disease.

### 3.2 Drug-induced liver injury

Drug-induced liver injury (DILI) is a major clinical challenge, arising from diverse agents including pharmaceuticals, herbal remedies, and dietary supplements. In severe cases, DILI may lead to acute liver failure, or even death (Björnsson and Björnsson, 2022). Among the various agents implicated in hepatotoxicity, acetaminophen (APAP) is one of the most commonly reported and extensively investigated synthetic compounds. The primary mechanism underlying its hepatocellular toxicity involves the formation of reactive metabolites during hepatic metabolism, which induce severe oxidative stress, inflammatory responses, and hepatocellular apoptosis (Rani et al., 2024).

Emerging evidence indicates that the Notch signaling pathway plays a crucial regulatory role in cell fate determination and tissue regeneration following DILI. In APAP-induced liver injury (AILI), the Notch pathway exerts protective effects across multiple cell types. In macrophages, deletion of phosphatase and tensin homolog (PTEN) activates Notch1 signaling, which enhances the nuclear interaction between NICD and NRF2, thereby inhibiting the STING pathway and alleviating inflammation and necrosis (Yang et al., 2023). Additionally, in mesenchymal stem cells, Jagged1-mediated Notch2 activation upregulates COX2/PGE2 signaling, which subsequently inhibits the NLRP3 inflammasome via the AMPK/SIRT1 axis in macrophages to attenuate liver injury (Yu et al., 2022).

Although numerous studies indicated that various drugs can induce liver injury by dysregulating the Notch signaling pathway, it is noteworthy that certain pharmacological agents can exert hepatoprotective effects through targeted modulation of this pathway. For instance, matrine has been shown to alleviate liver injury and promote hepatic regeneration by suppressing the hyperactivated Notch pathway and facilitating the differentiation of hepatic oval cells into functional hepatocytes (Shi et al., 2020).

Furthermore, Aflatoxin B1, a potent carcinogen produced by *Aspergillus flavus*, induces hepatotoxicity closely associated with intestinal barrier dysfunction. It activates the intestinal Notch signaling pathway, upregulates Hes1 expression, and inhibits goblet cell differentiation, thereby compromising the intestinal barrier. This disruption weakens intestinal integrity and contributes indirectly to inflammation and exacerbated liver injury. Conversely, Penthorum chinense Pursh compound flavonoids inhibit the Notch pathway, reduce Hes1 expression, restore the intestinal barrier, and improves the microbiota structure, thereby mitigating Aflatoxin B1-induced liver damage (Tao et al., 2023).

Collectively, these findings highlight the dual role of the Notch signaling pathway in DILI, functioning either as a protective mechanism or as a contributor to tissue damage, which underscores its potential as a therapeutic target for both preventing hepatotoxicity and enhancing hepatic repair processes. Furthermore, the temporal dynamics of pathway activation—during initial injury versus recovery phases—critically influence its protective versus detrimental effects. Understanding these contextual determinants provides a framework for developing targeted Notch-based therapies that can be precisely timed and cell-directed to maximize protective outcomes while minimizing potential adverse effects in DILI management.

### 3.3 Hepatic ischemia-reperfusion injury

Hepatic ischemia-reperfusion injury (HIRI) is a frequent complication following extensive hepatic resection and involves pathological mechanisms such as oxidative stress, inflammation, and mitochondrial dysfunction, all of which can lead to significant liver damage (de Oliveira and Gonçalves, 2025). During the pathological process of HIRI, the Notch signaling pathway exerts multidimensional regulatory effects across different cell types through specific molecular mechanisms.

In liver sinusoidal endothelial cells (LSECs), Notch signaling activation has been shown to downregulate the expression of endomucin (EMCN), a key glycoprotein involved in maintaining endothelial barrier integrity. This downregulation promotes neutrophil adhesion and transendothelial migration mediated by LFA-1, thereby exacerbating inflammatory responses. Notably, experimental evidence from endothelial-specific Notch activation mouse models demonstrates a marked reduction in EMCN levels. Conversely, RBPj deletion or Notch signaling inhibition reverses this phenomenon, confirming the critical role of the Notch-EMCN axis in regulating neutrophil infiltration (Zhang et al., 2020). Furthermore, in macrophages, Notch1 exerts an anti-inflammatory effect by activating the β-catenin signaling pathway. This interaction ultimately suppresses TRAF6/TAK1-mediated innate immune responses and RIPK3/MLKL-dependent hepatocyte necroptosis. Notch1 deficiency leads to decreased β-catenin expression, enhanced TAK1 activity, and increased release of inflammatory factors. Importantly, restoration of β-catenin expression reverses this damage, revealing the pivotal role of the Notch1–β-catenin axis in regulating hepatic inflammation and cell death (Xu et al., 2022). Additionally, in myeloid cells, JAG1-mediated Notch1 signaling exerts protective effects by activating the HSF1/Snail axis. This pathway upregulates thioredoxin, inhibits TXNIP/NLRP3/caspase-1 inflammasome activation, and reduces the release of pro-inflammatory cytokines such as interleukin-1β (IL-1β) (Jin et al., 2020). Interestingly, Rehmannia glutinosa glycoside has been demonstrated to delay cellular senescence in LSECs by inhibiting the LRP1-Notch1-C/EBPβ signaling axis. This intervention blocks Notch1-mediated binding of C/EBPβ to the Il-1b promoter, thereby suppressing the pro-inflammatory effects of the senescence-associated secretory phenotype (Zhang et al., 2024b). Moreover, exosomes derived from mesenchymal stem cells preconditioned with transforming growth factor-beta 1 (TGF-β1) enhance therapeutic efficacy by activating the JAG1/Notch1/SOX9 pathway, which significantly alleviates injury in biliary epithelial cells by reducing oxidative stress and apoptosis (Yasen et al., 2023). In conclusion, Notch signaling exhibits cell-specific dual regulatory roles in HIRI, either exacerbating injury or promoting protection depending on the cellular origin and the precise temporal and pathological context of its activation. This mechanistic understanding lays a critical theoretical foundation for developing precise targeted therapeutic strategies.

### 3.4 NAFLD

**FIGURE 1:**
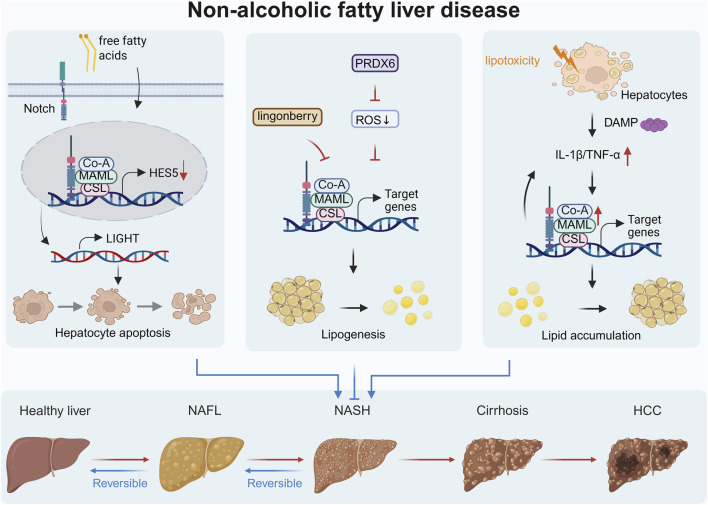
NAFLD encompasses a series of pathological changes, including NAFL, NASH, liver fibrosis, liver cirrhosis, and eventually may progress to HCC. The abnormal activities of notch signaling pathway constitute the core mechanism of the progression of NAFLD. (Figure created using BioRender.com).

NAFLD is a disease spectrum closely related to insulin resistance and genetic susceptibility, characterized by excessive hepatic lipid accumulation without excessive alcohol consumption or other clear liver damage factors (Chen et al., 2022; Steinman et al., 2021). Emerging evidence reveals that in the context of chronic liver damage such as NASH, some highly conserved signaling pathways that determine cell fate during embryonic liver development are reactivated. The dysregulation of these pathways collectively constitutes the core mechanism of the progression of NAFLD (Feng et al., 2022). Among these pathways, the aberrant activation of the Notch signaling pathway plays a multifaceted role in the onset and progression of NAFLD (Figure 1). Its effects are mediated through various cell types within the liver, contributing to metabolic dysregulation, inflammation, and microenvironment disruption.

At the hepatocyte level, dysregulated lipid metabolism can lead to Notch signaling dysfunction. Research indicates that free fatty acids downregulate the expression of HES5, a downstream transcription factor of the Notch pathway, thereby relieving its transcriptional inhibition on the pro-apoptotic factor LIGHT and exacerbating hepatocyte apoptosis. This mechanism reveals the key role of the Notch/HES5 axis in hepatocyte injury in NAFLD (Miao et al., 2021). Conversely, lingonberry extract has demonstrated protective effects by attenuating hepatic lipid accumulation through inhibition of Notch1 signaling, which downregulates SREBP-1c-mediated lipogenesis and promotes fatty acid oxidation (Madduma Hewage et al., 2022). Further studies reveal that PRDX6 deficiency enhances oxidative stress-induced activation of Notch signaling, while inhibition of Notch improves lipid deposition and mitigates mitophagy, suggesting that Notch plays a key role in the vicious cycle between oxidative stress and dysregulated lipid metabolism (Lee et al., 2019).

Beyond hepatocytes, the Notch pathway contributes to NAFLD pathogenesis by regulating non-parenchymal cells, thereby promoting inflammation and microenvironment imbalance. In LSECs, abnormal activation of Notch inhibits the transcription of endothelial nitric oxide synthase, leading to LSEC dysfunction and exacerbating hepatic microcirculatory disturbances and inflammatory responses (Fang et al., 2022). Similarly, in macrophages, the Notch pathway is upregulated via RBP-J-dependent transcriptional mechanisms, thereby promoting the release of pro-inflammatory cytokines such as IL-1β and TNF-α and ultimately amplifies the inflammatory cascade. Notably, targeted intervention using exosomes to deliver RBP-J “decoy” oligodeoxynucleotides effectively inhibits Notch signaling in macrophages and improves NAFLD phenotypes, thus demonstrating the therapeutic potential of targeting this pathway (Ding et al., 2023).

In conclusion, the Notch signaling pathway is involved in the pathological processes of NAFLD, including hepatocyte lipid metabolism, oxidative stress, endothelial function, and immune inflammation. The cell-specific manifestations of Notch signaling in NAFLD—ranging from metabolic regulation in hepatocytes to inflammatory activation in immune cells—exemplify how cellular origin and microenvironmental cues critically determine its functional outcomes. Therapeutic interventions targeting Notch signaling present promising avenues for NAFLD treatment, particularly through cell-type-specific modulation strategies that account for the pathway’s context-dependent functionality.

### 3.5 Liver fibrosis

**FIGURE 2:**
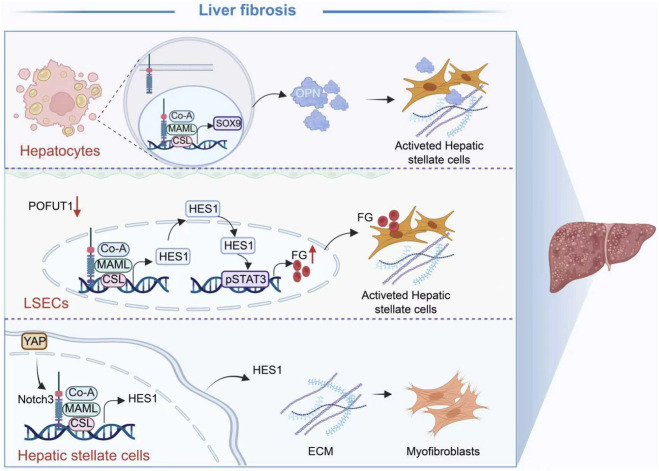
The Notch pathway plays a central role in the development of liver fibrosis by coordinating between parenchymal cells and non-parenchymal cells. (Figure created using BioRender.com).

The pathogenesis of liver fibrosis involves complex intercellular communication and intracellular signaling reprogramming. The Notch signaling pathway, a critical regulator of cell fate and function, plays a central role in driving the profibrogenic microenvironment by coordinating a intricate network of parenchymal and non-parenchymal cells (Figure 2).

#### 3.5.1 Role of hepatocyte notch signaling in fibrosis

As the primary functional units of the liver, hepatocytes serve as a critical bridge connecting metabolic stress to fibrosis through aberrant activation of Notch signaling. In the context of NASH, insults such as lipotoxicity activate the Toll-like receptor 4 (TLR4)-NF-κB axis in hepatocytes, significantly upregulating the expression of JAG1 and thereby initiating Notch signaling (Yu et al., 2021). Activated Notch signaling in hepatocytes drives the fibrogenic process through multiple downstream pathways. For example, the activation of the Notch pathway induces the expression of the transcription factor Sox9, leading to the upregulation and secretion of Osteopontin (OPN), which in turn activates HSCs in a paracrine manner (Zhu et al., 2018). Additionally, the downstream effector of Notch signaling, Ephrin type B receptor 2, has been demonstrated to be sufficient to induce cell-autonomous inflammation in hepatocytes, exacerbating local injury (Xiao et al., 2023). Furthermore, studies reveal that Notch signaling can directly bind to and transactivate the promoter of the Monocyte Chemoattractant Protein-1 (MCP-1/CCL2) gene, resulting in substantial MCP-1 secretion. This recruits monocyte-derived macrophages to the liver, amplifying inflammation and fibrogenesis (Kang et al., 2023). Recent research has also revealed that the Hippo pathway effectors YAP/TAZ form a complex with TEAD to upregulate the expression of the transcription factor CHCHD2. This subsequently amplifies Notch signaling in hepatocytes and stimulates the production of downstream OPN, thereby forming a complete profibrogenic axis to activate HSCs (Li et al., 2022).

#### 3.5.2 Role of LSEC notch signaling in fibrosis

Capillarization of LSECs represents an early event in the initiation of liver fibrosis. Precise regulation of Notch signaling within LSECs is crucial for maintaining their homeostasis. Research indicates that endothelial-specific deletion of POFUT1, a key glycosyltransferase essential for Notch signaling, disrupts Notch signaling homeostasis, thereby enhances the Notch/HES1/STAT3 signaling axis and leading to a significant upregulation of fibrinogen expression. This aberrantly produced and secreted fibrinogen from LSECs acts as a potent paracrine signal that directly activates neighboring HSCs, driving their activation and proliferation and initiating the fibrogenic process (He et al., 2024).

#### 3.5.3 Notch signaling in HSCs and myofibroblasts

HSCs are the primary cells responsible for extracellular matrix production, and their activation is central to fibrogenesis. The Notch signaling pathway plays a dual role within HSCs and their terminally differentiated myofibroblasts (MFs), functioning as both a direct driver and a dynamic regulator. During fibrosis progression, activation of the Notch3 receptor and its downstream effector Hes1 in HSCs/MFs directly promotes their activation and fibrogenesis. Notably, the activation of Notch3 is subject to upstream regulation by YAP, a key effector of the Hippo pathway. However, YAP inhibition downregulates Notch3 expression, highlighting the core intrinsic mechanism of the YAP-Notch3 axis in driving HSC activation (Yang et al., 2022). Furthermore, studies during fibrosis regression show that specific blockade of Notch signaling in MFs not only increases the expression of matrix metalloproteinases (MMPs) to enhance ECM degradation but also upregulates Hepatocyte Growth Factor (HGF) to promote hepatocyte proliferation. Concurrently, it induces the expression of pro-apoptotic factors, thereby accelerating MF apoptosis. This demonstrates the bidirectional regulatory role of Notch signaling in MFs during fibrosis progression and regression (Yue et al., 2021).

#### 3.5.4 Role of notch signaling in the hepatic immune microenvironment

Notch signaling is also deeply involved in modulating the hepatic immune microenvironment, indirectly influencing fibrosis. A recent study shows that activation of RBP-J is of importance in regulating macrophage activation and plasticity, thereby influencing the inflammation-fibrosis axis (He et al., 2022). Additionally, in the unique pathology of ALGS, dysregulated Notch signaling affects hepatocyte maturation and immune cell function, which collectively determines a distinct fibrotic phenotype different from other cholestatic liver diseases. These findings further highlight the complexity of Notch-mediated immune regulation (Mašek et al., 2024).

In summary, the Notch signaling pathway constructs a highly sophisticated and complex regulatory network in liver fibrosis. It not only drives fibrosis through cell-intrinsic mechanisms within the three core cell types (hepatocytes, LSECs, and HSCs/MFs), but also influences the microenvironment by regulating the function of immune cells like macrophages and dynamically controlling the balance between fibrosis progression and regression. The cell-type-specific actions and microenvironmental influences detailed in this section exemplify how cellular origin and microenvironmental cues critically shape the pro-fibrotic functions of Notch signaling. A deep understanding of the specific mechanisms of the Notch pathway within this complex network provides a crucial perspective for comprehensively elucidating the pathogenesis of liver fibrosis.

## 4 Notch signaling pathway in liver regeneration

The liver is recognized as an organ with high regenerative capacity. While trans-differentiation between hepatocytes and cholangiocytes is known to occur following severe chronic liver injury, Notch signaling has been established as a key regulator of liver regeneration, critical for maintaining hepatic homeostasis and driving the conversion of hepatocytes into hepatocytes into cholangiocyte-like cells in response to injury (Huang et al., 2022; Zhang et al., 2025b).

In acute liver injury models, transient early activation of Notch1-and Notch3-mediated signaling is essential for BECs to enter the cell cycle and undergo proliferative expansion. This process depends on Notch signaling upregulating the expression of Insulin-like Growth Factor 1 Receptor within BECs, although the expansion of BECs and their differentiation into hepatocytes are governed by distinct mechanisms (Minnis-Lyons et al., 2021). Moreover, transitional liver progenitor cells (TLPCs) originate from BECs and possess bipotent differentiation potential, allowing them to further differentiate into hepatocytes or revert to a BEC fate. Notch signaling primarily drives the initial conversion of BECs to TLPCs, whereas the subsequent differentiation and maturation of TLPCs into functional hepatocytes are predominantly governed by Wnt/β-catenin signaling (Pu et al., 2023). Interestingly, in a zebrafish model, the Farnesoid X Receptor was shown to specifically promote the redifferentiation of bipotential progenitor cells into BECs by activating Notch signaling, while differentiation into hepatocytes is mediated by ERK1 signaling. This highlights the specific function of Notch signaling in the fate decision within the biliary epithelial lineage for progenitor cells (Cai et al., 2021).

Additionally, following partial hepatectomy or acute injury, compensatory proliferation of remaining hepatocytes is the primary mode of regeneration. LSECs play a crucial role in regulating hepatocyte proliferation. Duan et al. have identified a specific c-Kit-positive LSEC subset that promotes hepatocyte proliferation by secreting Wnt2. However, activation of Notch signaling alters the spatial distribution and inhibits the function of this subset, thereby diminishing pro-regenerative signals and impeding liver regeneration (Duan et al., 2022b). Significantly, sustained activation of Notch in endothelial cells during later stages of regeneration leads to transcriptional repression of the deacetylase Sirtuin 1 by its downstream effector Hes1. This process induces LSEC senescence along with a senescence-associated secretory phenotype, which disrupts sinusoidal remodeling and ultimately inhibits the regenerative process (Duan et al., 2022a).

In immune-mediated liver injury, infiltrating monocyte-derived macrophages (MoMFs) activate the Notch2 receptor on hepatocytes surrounding the lesion via their surface Jagged1 (JAG1) ligand. This interaction induces the expression of SRY-box transcription factor 9 (SOX9), leading to the transformation of the MoMFs into a cell death-resistant phenotype. This process ultimately forms a protective barrier that confines the necrotic area (Feng et al., 2023). Additionally, it is demonstrated that transplantation of human umbilical cord-derived mesenchymal stem cells can improve liver function and promote liver regeneration by inhibiting the Notch signaling pathway, indicating that modulating Notch activity is a significant mechanism of regenerative therapies (He et al., 2021).

In conclusion, Notch signaling constructs a multi-layered, highly context-dependent regulatory network in liver regeneration. The pathway’s capacity to either facilitate or impede regeneration depending on specific cellular contexts and injury phases highlights its context-dependent functionality. A deep understanding of the function of Notch signaling in specific cells and spatiotemporal contexts is crucial for developing therapeutic strategies targeting the regenerative process.

## 5 Notch signaling pathway in liver cancer

Sustained activation of Notch signaling is linked to hepatic malignancies, including HCC with stem cell characteristics and intrahepatic cholangiocarcinoma (ICC) (Geisler and Strazzabosco, 2015).

Numerous studies have demonstrated that Notch signaling acts as a canonical oncogenic driver in the development and progression of ICC. However, in HCC, its function can be either a tumor suppressor or transformed into an oncogenic factor under specific conditions (D'Assoro et al., 2022; Fang et al., 2023). Critical interpretation of these seemingly contradictory findings must take into account the specific experimental models used, as the choice of cell lines and animal models can significantly influence the observed outcomes. Therefore, a thorough dissection of the context-specific mechanisms of Notch signaling in HCC and ICC is essential for understanding the heterogeneity of liver tumors and developing precise targeted therapeutic strategies. The following sections will systematically elucidate the complex roles of the Notch signaling pathway in liver cancers, with separate discussions focused on HCC and ICC (Table 2).

**TABLE 2:** Notch signaling pathway in liver cancer.

Cancer	Notch signaling component	Role	Mechanism	Cell line	Animal model	Reference
ICC	Notch1	Oncogene	Notch1 activation cooperates with YAP → upregulates amino acid transporters and GLS1 → activates mTORC1 signaling → promotes cholangiocarcinogenesis	Normal human cholangiocytes (NHC-SS and C324)	Wild-type FVB/N mice and Raptorfl/fl mice	Lu et al. (2021)
ICC	Notch 2, NICD2	Oncogene	MANF upregulation → cytosolic CK19 binds to AR domain of NICD2 → activates Notch2 nuclear signaling → drives hepatocyte reprogramming into ICC cells	Hucct1, HCCC-9810, RBE cells	SBT- and TAA-induced mouse ICC models, hepatocyte-specific MANF knockin/knockout mice, subcutaneous xenograft model	Mei et al. (2025)
ICC	Notch 1, Notch 2	Oncogene	TAZ activation → induces Notch pathway activation → drives biliary commitment via SOX9 → determines cholangiocellular phenotype of tumors	RBE, KKUM-213, and HuccT1 human iCCA cell lines	FVB/N mice hydrodynamic injection model of TAZ/AKT plasmids	Cigliano et al. (2022)
ICC	Notch 1, Notch 2, NICD	Oncogene	Numb loss → fails to bind and degrade NICD → enhances Notch signaling activation → promotes hepatic progenitor expansion and malignant transformation	—	DDC diet-induced HPC expansion model, TAA-induced iCCA model	Shu et al. (2021)
ICC	Notch 1, Notch 4, HES1	Oncogene	Notch signaling activation→ upregulates HES1 expression → HES1 promotes CFL1and ID1 transcription → drives cholangiocyte lineage commitment	—	C57BL/6J mice hydrodynamic injection model of AKT/NICD plasmids	Wang et al. (2022)
ICC	Notch	Oncogene	Notch activation → promotes TGF-β1/Smad2 signaling in CAFs → resulting in a fibrotic tumor microenvironment	—	PDX model and hydrodynamic ICCA model (AKT/Jagged1)	Mancarella et al. (2022a)
ICC	Notch1, HES1	Oncogene	Notch1 → promotes THY1/CD90 expression → enhances tumor stemness and activates PI3K/AKT signaling → promotes aggressive ICC phenotype	HuCCT1, RBE, KKU-M213, and KKU-M156 human iCCA cell lines	PDX model	Mancarella et al. (2022b)
ICC	Notch1	Oncogene	PKHD1↓→ Notch Pathway activation → enhances EMT → enhances ICC proliferation/invasion/migration	Human ICC cell lines HCCC-9810, RBE, IHC-ST1, HuCCT1	BALB/c nude mice subcutaneous xenograft model	Shang et al. (2024)
HCC	Notch1, NICD1, Hes1	Oncogene	UBE2C overexpression → activates Notch1 signaling → induces EMT→ promotes HCC proliferation and metastasis	PLC/PRF/5 cells, HEK293T, Huh7 and Hep3B cells	BALB/c nude mice subcutaneous xenograft, tail vein injection metastasis models	Zhan et al. (2024)
HCC	Notch	Oncogene	SORT1 overexpression → activation Notch signaling pathway → increase in CD133 expression → promotion of HCC progression	THLE-2 normal liver cell line, Human HCC cell lines Huh-7, Hep3B, PLC/PRF/5, SNU368, SNU398, SNU423, SNU449, and SNU475	—	Ahn et al. (2024)
HCC	Notch1, Notch 2	Oncogene	TBC1D15 binds to Notch1 → Blocks CDK8/CDK19-mediated phosphorylation and FBW7 recruitment → promotes self-renewal and maintenance of TICs	Huh7, Hep3B, Hepa1-6 cells	Genetically engineered mouse models	Choi et al. (2024)
HCC	Notch1	Oncogene	Hypoxia/HIF-1α → transcriptional activation of DTL → ubiquitination of SLTM → activation Notch1 → promotes HCC proliferation, metastasis, and sorafenib resistance	Huh-7, HCCLM-3, and HEK-293T cells	Tumor xenograft models	Chen et al. (2024b)
HCC	Notch2	Oncogene	SCAFs → SOX4 upregulates CTHRC1 → activates Notch1 signaling pathway → promotes cancer stemness and metastasis	SNU-398 cell line, MHCC-97 H hepatocellular carcinoma and HEK-293T human embryonic kidney cell lines	BALB/c nude mice orthotopic liver xenograft model	Huang et al. (2025)
HCC	JAG1	Oncogene	AID interacts with HAT1 → activates Notch signaling → induces c-FOS expression → promotes HCC metastasis	293T cells, LO2, HepG2, SMMC-7721, HCC-LM3, and MHCC-97L HCC cell lines	—	Jiao et al. (2025)
HCC	Notch	Oncogene	CLDN4 palmitoylation → recruits CNTN1 → activates Notch signaling pathway → induces HBT→ promotes lenvatinib resistance	MHCC97H, PLC/PRF/5, Huh7, Hepa1-6 cells	Spontaneous HCC mouse model, orthotopic tumor model, subcutaneous tumor model	Xu et al. (2025)
HCC	Notch1	Oncogene	IGFBP4 → inhibits the Notch1 signaling pathway → inhibits EMT and metastasis	Human liver cancer cell lines HCCLM3, Huh7, HepG2	Tumor xenograft models	Sun et al. (2025)
HCC	Notch	Oncogene	NELL2→ inhibits the Notch1 signaling pathway → inhibits EMT	Normal liver cell line LO2, liver cancer cell lines Huh-7, Bel-7402, SK-Hep-1, HepG-2, and HCC-LM3	—	Liu et al. (2025b)
HCC	Notch1	Oncogene	Notch1+CD8+T phenotype → impairs T cell cytotoxicity and cytokine secretion → poor response to immunotherapy	—	—	Pu et al. (2024)
HCC	NICD	Oncogene	SLFN11 binds competitively with TRIM21→ inhibits RBM10 ubiquitination → inhibits Notch pathway activation → enhances anti-tumor immunity	SLFN11-knockdown SMMC-7721 cells	Humanized mice orthotopic liver cancer model	Zhou et al. (2023a)
HCC	Notch1, JAG	Oncogene	Lenvatinib treatment → Notch1 & JAG ↑ → Increases cancer stem cell marker CD44 → promotes Lenvatinib resistance	Hep3B cells	—	Feng et al. (2025)
HCC	Notch1	Oncogene	OBB → inhibiting the Notch1-USP7-c-Myc axis → enhancing the sensitivity to sorafenib	Hep3B, Huh7 cell line, human liver cancer cell line LM3	BALB/c nude mice subcutaneous xenograft model	Sun et al. (2024b)
HCC	JAG1, HEY1	Oncogene	FNC binds to JAG1 → Inhibits Notch signaling pathway → suppresses HCC migration	Huh7 cells, HepG2 and PLC/PRF/5 cells	—	Meng et al. (2025)
HCC	Notch1	Oncogene	Synergistic interplay between Pin1 and Notch1 → maintains CSC aggressiveness and stemness → promotes radioresistance	HepG2, Bel7402, and MHCC97H cells	Subcutaneous HCC Models, PDX Models	Cao et al. (2025)
HCC	Notch2	Tumor Suppression	Activation of Notch1 → induces G0/G1 cell cycle arrest → downregulates cyclins → inhibits HCC cell growth *in vitro* and *in vivo*	Human HCC cells SMMC7721, and 293T cells	—	Qi et al. (2003)
HCC	Notch	Tumor Suppression	RB pathway inactivation & tumor progression → E2F activation → activates Notch pathway → slows HCC growth	HepG2, SNU-449, Hep3B cells, primary mouse HCC cells	Genetically engineered mouse models	Viatour et al. (2011)
HCC	Notch1	Tumor Suppression	HBx protein → suppresses presenilin1 transcription → inhibits Notch1 → promotes cell proliferation, G1-S progression, and blunts cellular senescence → facilitates HCC	Huh7, Hep3B, and HepG2	Tumor xenograft models	Xu et al. (2010)
HCC	HES5	Tumor Suppression	HES5 ↑ → inhibits HES1 & downregulates MYC targets (ODC1, LDHA) → suppresses MYC-dependent tumorigenesis	HEK293T cells, immortalized hepatocyte cell line HHT4, liver cancer cell lines HuH1, HuH7, SNU182, SNU475, HepG2, Hep3B, HLE, HLF, PLC, KMCH1, and HUCCT1	MYC/AKT-driven mouse HCC models	Luiken et al. (2020)
HCC	Notch2, JAG1	Tumor Suppression	JAG1 ↑ in mesenchymal cells → activates Notch2 → suppresses Dll4/Notch1 signaling → inhibits tumor progression	HCC cells	DEN-induced mouse HCC model	Nakano et al. (2022)

### 5.1 ICC

ICC originates from the bile duct epithelium. It is an almost universally fatal cancer with a dismal prognosis, and the scarcity of effective treatments particularly molecular targeted therapies, underscores it as a critical unmet medical need (Li et al., 2020). Notch signaling plays a well-defined role as an oncogenic driver in ICC. Studies demonstrate a significant synergistic effect between the Notch pathway and the Hippo pathway effector YAP. Their co-activation upregulates amino acid transporters, subsequently activating the mTORC1 signaling pathway, thereby potently driving ICC tumorigenesis (Lu et al., 2021).

At the cellular origin level, Notch signaling is a key mediator in the transdifferentiation of hepatocytes into ICC. Mesencephalic astrocyte-derived neurotrophic factor (MANF) triggers Notch signaling by stabilizing the NICD2, thereby directly facilitating the transformation of mature hepatocytes into ICC cells (Mei et al., 2025). Furthermore, in carcinogenesis driven by the Hippo effector TAZ, while Notch signaling is not essential for tumor initiation, it is critical for maintaining the cholangiocellular phenotype of the tumor cells (Cigliano et al., 2022).

Moreover, the loss of the tumor suppressor protein Numb promotes the expansion and malignant transformation of HPCs by relieving the inhibition on Notch signaling, further confirming the central role of Notch in determining ICC cell fate (Shu et al., 2021). Notably, Notch signaling indirectly promotes ICC progression by modulating the TME. Single-cell RNA sequencing analysis has revealed that periportal LSECs differentiate into tip endothelial cells that promote angiogenesis via the Dll4-Notch4-Efnb2 axis, thus supporting tumor growth (Wang et al., 2022). Investigation of spatial immunophenotypes in ICC found that the Notch signaling pathway is specifically enriched in the “excluded” immunophenotype characterized by immune cell restriction to the tumor margins, in which Notch activation promotes immune evasion (Zhu et al., 2023).

Concurrently, there exists a close association between Notch signaling and the activation of cancer-associated fibroblasts (CAFs). Inhibition of Notch significantly reduces TGF-β1 secretion and Smad-2 activation in CAFs, alleviating the extracellular matrix deposition and desmoplastic reaction mediated by CAFs. These findings indicate tight crosstalk between the Notch and TGF-β1 pathways (Mancarella et al., 2022b; Zhong et al., 2024). CD90 (THY1) has been identified as a direct transcriptional target downstream of Notch signaling. Its expression correlates with poor prognosis and may mediate cell-matrix interactions within the microenvironment (Mancarella et al., 2022b).

The activity of the Notch pathway is precisely regulated by various upstream factors. Research confirms that the polycystic kidney and hepatic disease 1 (PKHD1), a tumor suppressor gene, promotes ICC progression upon its downregulation by releasing the inhibition on Notch signaling, identifying PKHD1 as an important upstream negative regulator of the Notch pathway (Shang et al., 2024). Additionally, aberrant protein fucosylation enhances the interaction between NOTCH1 and its ligand Jagged1, which stabilizes and activates Notch signaling, thereby highlighting the role of post-translational modifications in pathway regulation (Ament et al., 2023).

From a clinical perspective, radiomics-based analysis shows enrichment of Notch signaling in aggressive ICC subtypes (Xin et al., 2024). Elevated expression of the Notch1/HES1/THY1 signaling axis is significantly associated with poor patient prognosis, indicating the important biomarker potential of the Notch pathway (Mancarella et al., 2022b).

Collectively, the Notch signaling pathway forms a complex oncogenic network in ICC, functioning both as a core driver of tumorigenesis and as a key regulator of the tumor microenvironment. Its pleiotropic effects—ranging from cellular transformation to immune microenvironment remodeling—are fundamentally shaped by specific receptor/ligand interactions and complex microenvironmental cues. The consistent observation of Notch hyperactivation across ICC models and patient specimens, coupled with its prognostic significance, solidifies its position as a compelling therapeutic target. Targeting upstream regulatory nodes or combination approaches addressing both tumor-intrinsic and microenvironmental functions of Notch may provide novel therapeutic opportunities for this aggressive malignancy.

### 5.2 HCC

**FIGURE 3:**
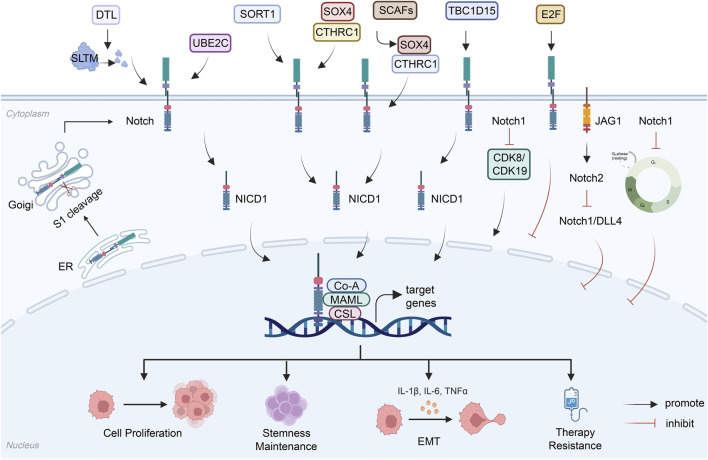
This schematic summarizes the dual functions of Notch signaling in HCC pathogenesis. While Notch frequently acts as an oncogenic driver by promoting tumor proliferation, stemness maintenance, EMT-driven metastasis, and therapy resistance through multiple upstream regulators and effector pathways, it can also exert tumor-suppressive effects under specific conditions. These include inducing cell cycle arrest, engaging negative feedback loops, and functioning through distinct ligand-receptor pairs such as Dll4/Notch1, with the net outcome determined by cellular context and molecular cues. (Figure created using BioRender.com).

The Notch signaling pathway plays a crucial role in promoting and inhibiting cancer development in HCC (Shi et al., 2024a; Yan et al., 2025). Its aberrant activation is driven by multiple mechanisms, including enhanced cell proliferation, induction of EMT, maintenance of stemness, and mediation of therapy resistance (Figure 3).

#### 5.2.1 Driving tumor cell proliferation and stemness maintenance

Notch signaling directly promotes HCC proliferation by regulating the cell cycle and stem cell properties. It has been demonstrated that UBE2C promotes HCC growth by upregulating N1ICD and Hes1, which are key components of the Notch pathway (Zhan et al., 2024). Additionally, SORT1 activates the Notch pathway to upregulate CD133 expression, enhancing the stemness characteristics of HCC cells (Ahn et al., 2024). Interestingly, at the level of tumor-initiating cells (TICs), TBC1D15 interacts with NOTCH1 to inhibit FBXW7-mediated ubiquitination and degradation of NICD1, thereby stabilizing NICD1 protein and promoting TIC self-renewal (Choi et al., 2024). A present study indicates that DTL promotes HCC proliferation by ubiquitinating and degrading SLTM, which relieves its transcriptional repression of Notch1 (Chen et al., 2024b). Furthermore, senescent cancer-associated fibroblasts (SCAFs) activate Notch1 signaling through the SOX4-CTHRC1 axis, promoting HCC stemness and metastasis (Huang et al., 2025). These findings collectively indicate that Notch pathway activation serves as a common downstream hub for various upstream factors driving HCC proliferation and stemness maintenance.

#### 5.2.2 Inducing EMT and promoting invasion and metastasis

Notch signaling plays a central role in EMT and metastasis in HCC. Research has revealed that AID forms a complex with HAT1, directly binds to the JAG1 gene promoter, regulates JAG1 transcription through histone modifications, and subsequently activates the Notch-c-FOS axis to drive HCC metastasis (Jiao et al., 2025). In the context of lenvatinib resistance, palmitoylated CLDN4 activates Notch signaling by recruiting contactin-1, inducing hepatic-to-biliary lineage transition (HBT) in HCC cells and enhancing their migratory and invasive capabilities (Xu et al., 2025). Moreover, emerging evidence demonstrates that IGFBP4 suppresses EMT and metastasis by inhibiting the Notch1 signaling pathway, while NELL2 impedes the EMT process by inhibiting Notch signaling (Liu et al., 2025b; Sun et al., 2025). These results confirm Notch signaling as a critical node in EMT regulation.

#### 5.2.3 Mediating therapy resistance

Activation of the Notch pathway is a key mechanism underlying therapy resistance in HCC. In the context of immunotherapy, Notch signaling activation is associated with CD8^+^ T cell exhaustion, leading tu diminished therapeutic response(Pu et al., 2024). However, SLFN11 enhances immunotherapy sensitivity by inhibiting Notch signaling(Zhou et al., 2023a). Regarding targeted therapy, lenvatinib induces cancer stem cell properties by activating Notch signaling, resulting in drug resistance (Feng et al., 2025). Conversely, OBB enhances sorafenib sensitivity by inhibiting the Notch1-USP7-c-Myc axis (Sun et al., 2024b). Additionally, Azvudine (FNC) directly binds to Jagged1, thereby inhibiting Notch signaling and reversing EMT-associated resistance (Meng et al., 2025). In radiotherapy, Pin1 cooperates with Notch1 to maintain cancer stem cell properties, leading to radioresistance (Cao et al., 2025).

#### 5.2.4 Tumor suppression mechanism

Although the Notch signaling pathway is more frequently reported to exert pro-tumorigenic effects in HCC, its tumor-suppressive functions have been increasingly revealed under specific molecular contexts and microenvironments. Accumulating evidence indicates that Notch1 signaling can mediate tumor-suppressive effects by inducing cell cycle arrest. Specifically, Notch1 activation downregulates Cyclin A/D1/E and CDK2 while upregulating p21 and p53 expression, leading to G0/G1 phase arrest in HCC cells and thereby inhibiting tumor growth (Qi et al., 2003). In HCC models with an inactivated Rb pathway, Notch pathway activation serves as a negative feedback mechanism that delays tumor progression via E2F transcription factors, and its activity level is positively correlated with patient prognosis (Viatour et al., 2011). The tumor-suppressive function of Notch1 is particularly significant in virus-associated HCC. The hepatitis B virus-encoded HBx protein has been demonstrated to inhibit presenilin1 transcription, resulting in a reduction in the cleavage-generated Notch1 intracellular domain, which ultimately inhibits Notch1 signaling. This inhibition leads to enhanced cell proliferation, accelerated cell cycle progression, and delayed cellular senescence, ultimately promoting HCC development, which conversely confirms the tumor-suppressive role of Notch1 in this context (Xu et al., 2010). At the molecular level, the Notch pathway target gene HES5 directly inhibits another Notch target gene HES1 and downregulates the pro-proliferative MYC targets ODC1 and LDHA, forming a negative feedback loop that suppresses cell migration and clonogenicity (Luiken et al., 2020). Notably, different Notch ligand-receptor pairs may exert opposing functions. Nakano and colleagues confirm that the Dll4/Notch1 signaling axis has a distinct tumor-suppressive role, as its specific knockout promotes tumor progression. Moreover, this axis exhibits an antagonistic relationship with the JAG1/Notch2 signaling axis (Nakano et al., 2022).

These findings demonstrate that the function of the Notch signaling pathway in HCC is highly context-dependent, with its tumor-suppressive effects contingent upon specific molecular environments, upstream regulatory mechanisms, and ligand-receptor interactions.

## 6 The treatment strategies targeting notch signaling for liver diseases

**FIGURE 4:**
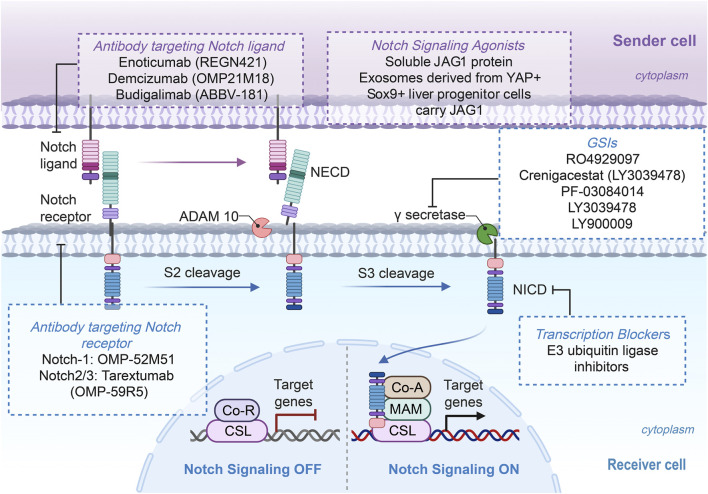
The Notch pathway plays a central role in the pathogenesis of liver diseases as a therapeutic target. The diagram outlines the core signaling process, highlighting potential intervention points that are currently under investigation. (Figure created using BioRender.com).

The Notch signaling pathway plays a central role in liver physiology and the pathogenesis of various liver diseases, making it a potential therapeutic target. Current therapeutic strategies targeting this pathway primarily include cleavage inhibitors, antibodies that specifically block ligand-receptor interactions, blockers of transcriptional activity, and agonists aimed at activating the pathway to promote tissue regeneration. The following section provides a detailed categorization of the key advancements (Figure 4). However, while cleavage inhibitors have advanced to early-phase clinical trials (Table 3), the majority of other strategies remain in the preclinical investigation stage (Shi et al., 2024b; Sun et al., 2024a; Zhou et al., 2022). Importantly, the clinical translation of these strategies, including those in early-phase trials, remains nascent. Challenges such as dose-limiting toxicities and modest efficacy underscore the necessity for continued preclinical optimization and the exploration of novel targets with improved therapeutic indices (BeLow and Osipo, 2020).

**TABLE 3:** Clinical trials of drugs targeting the Notch signaling pathway in liver cancer.

Type	Drugs	Cancer	Clinical trial ID	Phase	Status
GSI	RO4929097	Advanced Solid Tumors	NCT01218620	Phase 1	Completed
GSI	RO4929097	Unresectable Solid Malignancies	NCT01096355	Phase 1	Completed
GSI	Crenigacestat (LY3039478)	Advanced Solid Tumors	NCT02836600	Phase 1	Completed
GSI	BMS-986115	Advanced Solid Tumors	NCT01986218	Phase 1	Terminated
GSI	PF-03084014	Advanced solid malignancies	NCT00878189	Phase 1	Completed
GSI	LY3039478	Solid Tumors	NCT01695005	Phase 1	Completed
GSI	LY3039478	Solid Tumors	NCT02784795	Phase 1	Terminated
GSI	LY900009	Solid Tumors	NCT01158404	Phase 1	Completed
Notch1	OMP-52M51	Solid Tumors	NCT01778439	Phase 1	Completed
Notch2/3	Tarextumab (OMP-59R5)	Solid Tumors	NCT01277146	Phase 1	Completed
DLL4	Enoticumab (REGN421)	Advanced Solid Tumors	NCT00871559	Phase 1	Completed
DLL4	Demcizumab (OMP21M18)	Solid Tumors	NCT00744562	Phase 1	Completed
DLL3	Budigalimab (ABBV-181)	Solid Tumors	NCT03000257	Phase 1	Completed

### 6.1 γ-secretase inhibitors (GSIs)

As the key protease responsible for the final activation step of Notch receptors, γ-secretase represents a strategic intervention point, with its inhibitors (GSIs) capable of potently suppressing the entire signaling cascade (Fortini, 2002). It has been demonstrated that liver-specific Nicastrin antisense oligonucleotides (ASOs) or small-molecule GSIs ameliorate metabolic disorders through both Notch-dependent and Notch-independent mechanisms. The Notch-independent mechanism is of particular significance in this regard. By inhibiting γ-secretase-mediated cleavage of the low-density lipoprotein receptor (LDLR), it stabilizes LDLR protein levels, enhances hepatic uptake of triglyceride-rich lipoproteins, and effectively reduces plasma triglycerides (Kim et al., 2025). Furthermore, the GSI Dibenzazepine exhibits potent hepatoprotective effects in an APAP-induced acute liver injury model. Its mechanism involves the suppression of Notch-1 and Hes-1 expression, although there is a synergistic upregulation of autophagy-related proteins Beclin-1 and LC-3. This, in turn, leads to the alleviation of oxidative stress, inflammation, and apoptosis (Ahmed et al., 2021).

### 6.2 Antibodies against ligands or receptors

The objective of this strategy is to block the interaction between Notch ligands and receptors using specific antibodies, thereby enhancing the precision of targeting treatments. Investigations confirm that antibodies targeting Notch1 and Notch2, while showing expected effects in immune organs, failed to demonstrate antifibrotic efficacy in liver fibrosis models, suggesting the limitations of anti-Notch1/2 antibody therapy for fibrosis treatment. In contrast, the small-molecule inhibitor RIN1, targeting the key Notch transcriptional complex RBP-J has been shown to downregulate PD-L1 expression in tumor cells, which effectively alleviates CD8^+^ T cell exhaustion and enhances immunotherapy efficacy (Pan et al., 2023).

### 6.3 Transcription blockers

Research shows that the DHFR inhibitor Pralatrexate reduces methylation in the microRNA-34a promoter region to upregulate its expression, thereby inhibiting the Notch pathway and its downstream factors and ultimately enhancing the sensitivity of HCC cells to targeted agents such as sorafenib and lenvatinib (Jin et al., 2024). Additionally, E3 ubiquitin ligase inhibitors exert antitumor effects in HCC by promoting the degradation of the Notch1 NICD, which indirectly suppresses Notch signaling (Liu et al., 2019).

### 6.4 Notch signaling agonists

Under specific pathophysiological conditions, such as biliary injury repair or liver regeneration, moderate activation of Notch signaling may offer therapeutic benefits. A prior study indicated that in cholestatic liver injury, soluble JAG1 protein mimics the function of endogenous CCN1 protein by activating Notch signaling, thereby promoting cholangiocyte proliferation and ductular reaction. This process contributes to the repair of biliary damage and the enhancement of liver function (Kim et al., 2015). Additionally, a previous report revealed that during liver regeneration induced by portal vein ligation, exosomes derived from YAP + Sox9+ liver progenitor cells carry JAG1 and promote liver regeneration by activating Notch signaling in neighboring cells. This provides a novel strategy for intervening in regeneration by modulating exosome function (Liu et al., 2025a).

### 6.5 Multitarget peptide therapies and emerging strategies

Beyond classical strategies, emerging approaches involving multi-target interventions or regulation of endogenous signaling pathways demonstrate therapeutic potential. The kringle 1 domain of the hepatocyte growth factor α-chain (HGFK1), a multifunctional peptide, concurrently inhibits the Notch and Wnt/β-catenin pathways, thereby effectively reducing the CD90^+^ cancer stem cell (CSC) subpopulation and enhancing chemotherapy sensitivity (Li et al., 2024). Additionally, asiaticoside suppresses hepatic stellate cell activation and exerts antifibrotic effects by downregulating the expression of Notch1, JAG1, and DLL4(Xiao and Zhang, 2023). Moreover, the antifibrotic effect of Huang Qi Decoction depends on upregulating Numb protein (a negative regulator of Notch), thereby inhibiting the Notch signaling pathway (Xu et al., 2020).

Therapeutic strategies targeting the Notch pathway have become increasingly diverse, ranging from GSIs and antibodies to transcriptional regulation, agonists, and natural compounds, providing precise intervention options for various liver diseases. Future efforts are expected to focus on further elucidating the pathologically specific functions of Notch signaling to develop safer and more effective therapies.

## 7 Future perspectives

The future direction of Notch-targeted therapy will focus on the following cutting-edge techniques to achieve precise intervention with higher specificity and lower toxicity. Future therapies will move beyond simple “activation” or “inhibition” towards spatiotemporally precise regulation of the signaling pathway. For instance, utilizing light-controlled hydrogel systems or engineered exosomes to deliver Notch ligands has the potential to facilitate on-demand, localized activation of Notch signaling during liver regeneration or biliary repair, offering a new paradigm for regenerative medicine (Liu et al., 2025a). Furthermore, targeting the Notch pathway represents a strategy to reshape the immune microenvironment. Studies indicate that Notch signaling is deeply involved in T cell exhaustion, and targeting its RBP-J can downregulate PD-L1 and restore T cell function (Pan et al., 2023; Pu et al., 2024). This provides a strong rationale for a novel combination strategy integrating Notch inhibitors and immune checkpoint inhibitors to overcome resistance in hepatocellular carcinoma immunotherapy. It is imperative that future treatment strategies should target the Notch pathway to reshape the immune microenvironment. In specific pathological contexts, the activation of Notch signaling has been observed to exert protective effects. Using soluble JAG1 protein or modulating upstream matrix proteins to activate Notch can promote biliary proliferation and injury repair, providing a novel approach for treating cholestatic liver diseases (Kim et al., 2015; Sparling et al., 2016). Another promising direction involves multi-target interventions. The intervention of upstream regulatory factors has been identified as a novel strategy to avoid the toxicity associated with direct Notch inhibition. For example, targeting CDCA8 or using the multi-target peptide HGFK1 can synergistically inhibit multiple pro-oncogenic pathways like Notch and Wnt, providing a new avenue for targeting cancer stem cells (Li et al., 2024; Wu et al., 2023). Additionally, future clinical trials may require the implementation of biomarker-based patient stratification strategies. In order to identify the most suitable patients for targeted therapy, it is crucial to assess Notch pathway activity scores, JAG1 amplification status, or specific receptor subtype expression in tumor tissue, which is key to realizing precision medicine. Briefly, these directions collectively outline a new blueprint for the future of liver disease treatment.

## 8 Conclusion

The Notch signaling pathway serves as a central regulatory axis in liver physiological homeostasis and pathological processes. This review systematically elucidates the context-dependent function of this pathway. In chronic liver injury, fibrosis, and tumorigenesis, its aberrant activation primarily exerts pro-fibrotic and pro-oncogenic effects by driving processes such as HSC activation, EMT, and cancer stemness. In contrast, during acute injury repair and liver regeneration, its timely activation plays a protective and pro-repair role. Given the mechanistic diversity, therapeutic strategies targeting Notch have evolved from initial broad-spectrum GSIs to a diverse toolbox encompassing specific antibodies, transcriptional blockers, agonists, and multi-target natural compounds, enabling precise intervention for different liver diseases. However, toxicity, drug resistance, and limited understanding of its functional complexity remain prevailing challenges. Therefore, an in-depth understanding of the precise functions of Notch signaling within specific liver pathological microenvironments is the key prerequisite for its successful clinical translation into effective therapies.

## Glossary

AILI: APAP-induced liver injury
ALD: alcohol-related liver disease
ALGS: Alagille syndrome
APAP: Acetaminophen
ASO: antisense oligonucleotides
BECs: biliary epithelial cells
CAFs: cancer-associated fibroblasts
CSC: cancer stem cell
DILI: Drug-induced liver injury
EMCN: endomucin
EMT: epithelial-mesenchymal transition
GSIs: γ-secretase inhibitors
HBT: hepatic-to-biliary lineage transition
HCC: hepatocellular carcinoma
HGF: Hepatocyte Growth Factor
HGFK1: hepatocyte growth factor α-chain
HIRI: Hepatic ischemia-reperfusion injury
HPCs: hepatic progenitor cells
HSCs: Hepatic stellate cells
ICC: intrahepatic cholangiocarcinoma
IL-1β: interleukin-1β
LDLR: low-density lipoprotein receptor
LSECs: liver sinusoidal endothelial cells
MANF: Mesencephalic astrocyte-derived neurotrophic factor
MFs: Myofibroblasts
MMPs: matrix metalloproteinases
MoMFs: monocyte-derived macrophages
NAFLD: non-alcoholic fatty liver disease
NASH: non-alcoholic steatohepatitis
NICD: Notch intracellular domain
OPN: Osteopontin
PKHD1: polycystic kidney and hepatic disease 1
PTEN: phosphatase and tensin homolog
SCAFs: senescent cancer-associated fibroblasts
SOX9: transcription factor 9
TGF-β1: factor-beta 1
TICs: tumor-initiating cells
TLPCs: transitional liver progenitor cells
TLR4: Toll-like receptor 4
TRX1: thioredoxin

## References

[B1] Adams J. M. Jafar-Nejad H. (2019). The roles of notch signaling in liver development and disease. Biomolecules 9 (10), 608. 10.3390/biom9100608 31615106 PMC6843177

[B2] Ahmed L. A. Abd El-Rhman R. H. Gad A. M. Hassaneen S. K. El-Yamany M. F. (2021). Dibenzazepine combats acute liver injury in rats via amendments of Notch signaling and activation of autophagy. Naunyn Schmiedeb. Arch. Pharmacol. 394 (2), 337–348. 10.1007/s00210-020-01977-0 32984915

[B3] Ahn H. R. Kim S. Baek G. O. Yoon M. G. Kang M. Ng J. T. (2024). Effect of Sortilin1 on promoting angiogenesis and systemic metastasis in hepatocellular carcinoma via the Notch signaling pathway and CD133. Cell Death Dis. 15 (8), 634. 10.1038/s41419-024-07016-7 39209807 PMC11362463

[B4] Ament C. E. Steinmann S. Evert K. Pes G. M. Ribback S. Gigante I. (2023). Aberrant fucosylation sustains the NOTCH and EGFR/NF-κB pathways and has a prognostic value in human intrahepatic cholangiocarcinoma. Hepatology 78 (6), 1742–1754. 10.1097/hep.0000000000000322 36789652

[B5] Aoshima K. Tanimizu N. (2025). Lineage plasticity and reprogramming of epithelial cells during tissue injury and regeneration-lessons from the lineage plasticity of hepatocytes and cholangiocytes induced by liver injury. Regen. Ther. 29, 447–454. 10.1016/j.reth.2025.04.008 40487923 PMC12144912

[B6] BeLow M. Osipo C. (2020). Notch signaling in breast cancer: a role in drug resistance. Cells 9 (10), 2204. 10.3390/cells9102204 33003540 PMC7601482

[B7] Björnsson H. K. Björnsson E. S. (2022). Drug-induced liver injury: pathogenesis, epidemiology, clinical features, and practical management. Eur. J. Intern Med. 97, 26–31. 10.1016/j.ejim.2021.10.035 34772600

[B8] Cai P. Mao X. Zhao J. Nie L. Jiang Y. Yang Q. (2021). Farnesoid X receptor is required for the redifferentiation of bipotential progenitor cells during biliary-mediated zebrafish liver regeneration. Hepatology 74 (6), 3345–3361. 10.1002/hep.32076 34320243

[B9] Cao H. Wang Q. Niu Y. Wang S. Jia H. Wang D. (2025). Dual-targeted nanovesicles induced cancer stem-like cell differentiation to sensitize hepatocellular carcinoma radiotherapy. Adv. Sci. (Weinh) 12 (39), e02409. 10.1002/advs.202502409 40697115 PMC12533360

[B10] Chen Y. Gao W. K. Shu Y. Y. Ye J. (2022). Mechanisms of ductular reaction in non-alcoholic steatohepatitis. World J. Gastroenterol. 28 (19), 2088–2099. 10.3748/wjg.v28.i19.2088 35664038 PMC9134136

[B11] Chen L. Chen J. Lou J. Yu J. (2024a). Clinical and genetic characteristics of patients with Alagille syndrome in China: identification of six novel JAG1 and NOTCH2 mutations. Transl. Pediatr. 13 (12), 2144–2154. 10.21037/tp-24-301 39823011 PMC11732638

[B12] Chen Z. X. Mu M. Y. Yang G. Qi H. Fu X. B. Wang G. S. (2024b). Hypoxia-induced DTL promotes the proliferation, metastasis, and sorafenib resistance of hepatocellular carcinoma through ubiquitin-mediated degradation of SLTM and subsequent Notch pathway activation. Cell Death Dis. 15 (10), 734. 10.1038/s41419-024-07089-4 39384740 PMC11464529

[B13] Chen C. Du Y. Nie R. Wang S. Wang H. Li P. (2025). Notch signaling in cancers: mechanism and potential therapy. Front. Cell Dev. Biol. 13, 1542967. 10.3389/fcell.2025.1542967 40052152 PMC11882598

[B14] Choi H. Y. Zhu Y. Zhao X. Mehta S. Hernandez J. C. Lee J. J. (2024). NOTCH localizes to mitochondria through the TBC1D15-FIS1 interaction and is stabilized *via* blockade of E3 ligase and CDK8 recruitment to reprogram tumor-initiating cells. Exp. Mol. Med. 56 (2), 461–477. 10.1038/s12276-024-01174-6 38409448 PMC10907578

[B15] Cigliano A. Zhang S. Ribback S. Steinmann S. Sini M. Ament C. E. (2022). The Hippo pathway effector TAZ induces intrahepatic cholangiocarcinoma in mice and is ubiquitously activated in the human disease. J. Exp. Clin. Cancer Res. 41 (1), 192. 10.1186/s13046-022-02394-2 35655220 PMC9164528

[B16] D'Assoro A. B. Leon-Ferre R. Braune E. B. Lendahl U. (2022). Roles of notch signaling in the tumor microenvironment. Int. J. Mol. Sci. 23 (11), 6241. 10.3390/ijms23116241 35682918 PMC9181414

[B17] de Oliveira T. H. C. Gonçalves G. K. N. (2025). Liver ischemia reperfusion injury: mechanisms, cellular pathways, and therapeutic approaches. Int. Immunopharmacol. 150, 114299. 10.1016/j.intimp.2025.114299 39961215

[B18] Devarbhavi H. Asrani S. K. Arab J. P. Nartey Y. A. Pose E. Kamath P. S. (2023). Global burden of liver disease: 2023 update. J. Hepatol. 79 (2), 516–537. 10.1016/j.jhep.2023.03.017 36990226

[B19] Ding J. Xu M. Du W. Fang Z. Q. Xu H. Liu J. J. (2023). Myeloid-specific blockade of notch signaling ameliorates nonalcoholic fatty liver disease in mice. Int. J. Biol. Sci. 19 (6), 1941–1954. 10.7150/ijbs.80122 37063432 PMC10092768

[B20] Duan J. L. Ruan B. Song P. Fang Z. Q. Yue Z. S. Liu J. J. (2022a). Shear stress-induced cellular senescence blunts liver regeneration through Notch-sirtuin 1-P21/P16 axis. Hepatology 75 (3), 584–599. 10.1002/hep.32209 34687050

[B21] Duan J. L. Zhou Z. Y. Ruan B. Fang Z. Q. Ding J. Liu J. J. (2022b). Notch-regulated c-Kit-Positive liver sinusoidal endothelial cells contribute to liver zonation and regeneration. Cell Mol. Gastroenterol. Hepatol. 13 (6), 1741–1756. 10.1016/j.jcmgh.2022.01.019 35114417 PMC9046233

[B22] Fang Z. Q. Ruan B. Liu J. J. Duan J. L. Yue Z. S. Song P. (2022). Notch-triggered maladaptation of liver sinusoidal endothelium aggravates nonalcoholic steatohepatitis through endothelial nitric oxide synthase. Hepatology 76 (3), 742–758. 10.1002/hep.32332 35006626

[B23] Fang Z. Meng Q. Xu J. Wang W. Zhang B. Liu J. (2023). Signaling pathways in cancer-associated fibroblasts: recent advances and future perspectives. Cancer Commun. (Lond). 43 (1), 3–41. 10.1002/cac2.12392 36424360 PMC9859735

[B24] Feng J. Qiu S. Zhou S. Tan Y. Bai Y. Cao H. (2022). mTOR: a potential new target in nonalcoholic fatty liver disease. Int. J. Mol. Sci. 23 (16), 9196. 10.3390/ijms23169196 36012464 PMC9409235

[B25] Feng D. Xiang X. Guan Y. Guillot A. Lu H. Chang C. (2023). Monocyte-derived macrophages orchestrate multiple cell-type interactions to repair necrotic liver lesions in disease models. J. Clin. Invest 133 (15), e166954. 10.1172/jci166954 37338984 PMC10378165

[B26] Feng X. Ping J. Gao S. Han D. Song W. Li X. (2024). Novel JAG1 variants leading to alagille syndrome in two Chinese cases. Sci. Rep. 14 (1), 1812. 10.1038/s41598-024-52357-0 38245625 PMC10799942

[B27] Feng W. Zhang H. Yu Q. Yin H. Ou X. Yuan J. (2025). Study on the mechanism of notch pathway mediates the role of lenvatinib-resistant hepatocellular carcinoma based on organoids. Curr. Mol. Med. 25 (3), 343–352. 10.2174/0115665240268201231213095302 38213137

[B28] Ferrandino M. Cardiero G. Di Dato F. Cerrato Y. Vitagliano L. Mandato C. (2024). Association of very rare NOTCH2 variants with clinical features of alagille syndrome. Genes (Basel) 15 (8), 1034. 10.3390/genes15081034 39202394 PMC11353882

[B29] Fortini M. E. (2002). Gamma-secretase-mediated proteolysis in cell-surface-receptor signalling. Nat. Reviews. Mol. Cell Biology 3 (9), 673–684. 10.1038/nrm910 12209127

[B30] Fu L. Gu X. Lou N. Li J. Xue C. (2025). Current research of the Notch pathway in hepatocellular carcinoma. Eur. J. Med. Res. 30 (1), 402. 10.1186/s40001-025-02626-z 40394648 PMC12090635

[B31] Geisler F. Strazzabosco M. (2015). Emerging roles of notch signaling in liver disease. Hepatology 61 (1), 382–392. 10.1002/hep.27268 24930574 PMC4268103

[B32] Gong Z. Shang B. Chu Y. Chen X. Li Q. Liu K. (2019). Fibrotic liver microenvironment promotes Dll4 and SDF-1-dependent T-cell lineage development. Cell Death Dis. 10 (6), 440. 10.1038/s41419-019-1630-1 31165736 PMC6549170

[B33] He Y. Guo X. Lan T. Xia J. Wang J. Li B. (2021). Human umbilical cord-derived mesenchymal stem cells improve the function of liver in rats with acute-on-chronic liver failure *via* downregulating Notch and Stat1/Stat3 signaling. Stem Cell Res. Ther. 12 (1), 396. 10.1186/s13287-021-02468-6 34256837 PMC8278604

[B34] He F. Li W. N. Li X. X. Yue K. Y. Duan J. L. Ruan B. (2022). Exosome-mediated delivery of RBP-J decoy oligodeoxynucleotides ameliorates hepatic fibrosis in mice. Theranostics 12 (4), 1816–1828. 10.7150/thno.69885 35198075 PMC8825583

[B35] He S. Luo Y. Ma W. Wang X. Yan C. Hao W. (2024). Endothelial POFUT1 controls injury-induced liver fibrosis by repressing fibrinogen synthesis. J. Hepatol. 81 (1), 135–148. 10.1016/j.jhep.2024.02.032 38460791

[B36] He X. Ma J. Yan X. Yang X. Wang P. Zhang L. (2025). CDT1 is a potential therapeutic target for the progression of NAFLD to HCC and the exacerbation of cancer. Curr. Genomics 26 (3), 225–243. 10.2174/0113892029313473240919105819 40433415 PMC12107793

[B37] Huang Q. Li J. Zheng J. Wei A. (2019). The carcinogenic role of the notch signaling pathway in the development of hepatocellular carcinoma. J. Cancer 10 (6), 1570–1579. 10.7150/jca.26847 31031867 PMC6485212

[B38] Huang R. Zhang X. Gracia-Sancho J. Xie W. F. (2022). Liver regeneration: cellular origin and molecular mechanisms. Liver Int. 42 (7), 1486–1495. 10.1111/liv.15174 35107210

[B39] Huang H. Peng W. Zhou Q. Zhao Y. Liu L. Cui H. (2025). Senescent fibroblasts secrete CTHRC1 to promote cancer stemness in hepatocellular carcinoma. Cell Commun. Signal 23 (1), 379. 10.1186/s12964-025-02369-8 40855439 PMC12376455

[B40] Jiao J. Shao K. Liu Z. Liu L. Nie Z. Wu J. (2025). Epigenetic activation of JAG1 by AID contributes to metastasis of hepatocellular carcinoma. J. Biol. Chem. 301 (1), 108078. 10.1016/j.jbc.2024.108078 39675704 PMC11758938

[B41] Jin Y. Li C. Xu D. Zhu J. Wei S. Zhong A. (2020). Jagged1-mediated myeloid Notch1 signaling activates HSF1/Snail and controls NLRP3 inflammasome activation in liver inflammatory injury. Cell Mol. Immunol. 17 (12), 1245–1256. 10.1038/s41423-019-0318-x 31673056 PMC7784844

[B42] Jin Y. Liu Q. Sun B. Li X. Wu J. Lin Z. (2024). Pralatrexate represses the resistance of HCC cells to molecular targeted agents via the miRNA-34a/Notch pathway. Discov. Oncol. 15 (1), 709. 10.1007/s12672-024-01572-2 39585461 PMC11589030

[B43] Kang J. Postigo-Fernandez J. Kim K. Zhu C. Yu J. Meroni M. (2023). Notch-mediated hepatocyte MCP-1 secretion causes liver fibrosis. JCI Insight 8 (3), e165369. 10.1172/jci.insight.165369 36752206 PMC9977430

[B44] Kim K. H. Chen C. C. Alpini G. Lau L. F. (2015). CCN1 induces hepatic ductular reaction through integrin αvβ_5_-mediated activation of NF-κB. J. Clin. Invest 125 (5), 1886–1900. 10.1172/jci79327 25822023 PMC4463205

[B45] Kim J. E. Kim Y. Bae J. Yoon E. L. Kim H. S. Lee S. R. (2025). A novel 11β-HSD1 inhibitor ameliorates liver fibrosis by inhibiting the notch signaling pathway and increasing NK cell population. Arch. Pharm. Res. 48 (2), 166–180. 10.1007/s12272-025-01534-4 39954198

[B46] Kitade M. Kaji K. Nishimura N. Seki K. Nakanishi K. Tsuji Y. (2019). Blocking development of liver fibrosis augments hepatic progenitor cell-derived liver regeneration in a mouse chronic liver injury model. Hepatol. Res. 49 (9), 1034–1045. 10.1111/hepr.13351 30989766

[B47] Kodama Y. Hijikata M. Kageyama R. Shimotohno K. Chiba T. (2004). The role of notch signaling in the development of intrahepatic bile ducts. Gastroenterology 127 (6), 1775–1786. 10.1053/j.gastro.2004.09.004 15578515

[B48] Kohut T. J. Gilbert M. A. Loomes K. M. (2021). Alagille syndrome: a focused review on clinical features, genetics, and treatment. Seminars Liver Disease 41 (4), 525–537. 10.1055/s-0041-1730951 34215014

[B49] Koike N. Tadokoro T. Ueno Y. Okamoto S. Kobayashi T. Murata S. (2022). Development of the nervous system in mouse liver. World J. Hepatol. 14 (2), 386–399. 10.4254/wjh.v14.i2.386 35317173 PMC8891673

[B50] Kopan R. Ilagan M. X. (2009). The canonical Notch signaling pathway: unfolding the activation mechanism. Cell 137 (2), 216–233. 10.1016/j.cell.2009.03.045 19379690 PMC2827930

[B51] Lee D. H. Jung Y. Y. Park M. H. Jo M. R. Han S. B. Yoon D. Y. (2019). Peroxiredoxin 6 confers protection against Nonalcoholic fatty liver disease through maintaining mitochondrial function. Antioxid. Redox Signal 31 (5), 387–402. 10.1089/ars.2018.7544 31007045

[B52] Li F. Peiris M. N. Donoghue D. J. (2020). Functions of FGFR2 corrupted by translocations in intrahepatic cholangiocarcinoma. Cytokine Growth Factor Rev. 52, 56–67. 10.1016/j.cytogfr.2019.12.005 31899106

[B53] Li Y. Xiu W. Xu J. Chen X. Wang G. Duan J. (2022). Increased CHCHD2 expression promotes liver fibrosis in nonalcoholic steatohepatitis *via* Notch/osteopontin signaling. JCI Insight 7 (23), e162402. 10.1172/jci.insight.162402 36477358 PMC9746920

[B54] Li X. Yan X. Wang Y. Kaur B. Han H. Yu J. (2023a). The Notch signaling pathway: a potential target for cancer immunotherapy. J. Hematol. Oncol. 16 (1), 45. 10.1186/s13045-023-01439-z 37131214 PMC10155406

[B55] Li Y. Yu Y. Yang L. Wang R. (2023b). Insights into the role of oxidative stress in hepatocellular carcinoma development. Front. Biosci. Landmark Ed. 28 (11), 286. 10.31083/j.fbl2811286 38062825

[B56] Li T. Liu L. Li L. Yao X. Hu X. Cheng J. (2024). HGFK1 enhances the anti-tumor effects of angiogenesis inhibitors *via* inhibition of CD90+ CSCs in hepatocellular carcinoma. Pharm. (Basel) 17 (5), 645. 10.3390/ph17050645 38794215 PMC11125149

[B57] Liu C. Cheng X. Chen J. Wang Y. Wu X. Tian R. (2019). Suppression of YAP/TAZ-Notch1-NICD axis by bromodomain and extraterminal protein inhibition impairs liver regeneration. Theranostics 9 (13), 3840–3852. 10.7150/thno.33370 31281517 PMC6587347

[B58] Liu M. Zhu Y. Li Z. Yu Y. Wang D. Wang Y. (2025a). Exosomes from liver progenitor cells carrying JAG1 activate notch signaling to promote liver regeneration in PVL rats. Cell Death Dis. 16 (1), 609. 10.1038/s41419-025-07925-1 40796539 PMC12343779

[B59] Liu S. Wu H. Zhang P. Zhou H. Wu D. Jin Y. (2025b). NELL2 suppresses epithelial-mesenchymal transition and induces ferroptosis via notch signaling pathway in HCC. Sci. Rep. 15 (1), 10193. 10.1038/s41598-025-94669-9 40133552 PMC11937300

[B60] Lu X. Peng B. Chen G. Pes M. G. Ribback S. Ament C. (2021). YAP accelerates notch-driven cholangiocarcinogenesis via mTORC1 in mice. Am. J. Pathol. 191 (9), 1651–1667. 10.1016/j.ajpath.2021.05.017 34129844 PMC8420864

[B61] Luiken S. Fraas A. Bieg M. Sugiyanto R. Goeppert B. Singer S. (2020). NOTCH target gene HES5 mediates oncogenic and tumor suppressive functions in hepatocarcinogenesis. Oncogene 39 (15), 3128–3144. 10.1038/s41388-020-1198-3 32055024 PMC7142020

[B62] Madduma Hewage S. Au-Yeung K. K. W. Prashar S. Wijerathne C. U. B. O K. Siow Y. L. (2022). Lingonberry improves hepatic lipid metabolism by targeting Notch1 signaling. Antioxidants (Basel) 11 (3), 472. 10.3390/antiox11030472 35326122 PMC8944850

[B63] Mancarella S. Gigante I. Serino G. Pizzuto E. Dituri F. Valentini M. F. (2022a). Crenigacestat blocking notch pathway reduces liver fibrosis in the surrounding ecosystem of intrahepatic CCA viaTGF-β inhibition. J. Exp. Clin. Cancer Res. 41 (1), 331. 10.1186/s13046-022-02536-6 36443822 PMC9703776

[B64] Mancarella S. Serino G. Gigante I. Cigliano A. Ribback S. Sanese P. (2022b). CD90 is regulated by notch1 and hallmarks a more aggressive intrahepatic cholangiocarcinoma phenotype. J. Exp. Clin. Cancer Res. 41 (1), 65. 10.1186/s13046-022-02283-8 35172861 PMC8851853

[B65] Martinez Lyons A. Boulter L. (2021). The developmental origins of Notch-driven intrahepatic bile duct disorders. Dis. Model Mech. 14 (9), dmm048413. 10.1242/dmm.048413 34549776 PMC8480193

[B66] Martinez Lyons A. Boulter L. (2023). NOTCH signalling - a core regulator of bile duct disease? Dis. Model Mech. 16 (9), dmm050231. 10.1242/dmm.050231 37605966 PMC10461466

[B67] Mašek J. Filipovic I. Van Hul N. Belicová L. Jiroušková M. Oliveira D. V. (2024). Jag1 insufficiency alters liver fibrosis via T cell and hepatocyte differentiation defects. EMBO Mol. Med. 16 (11), 2946–2975. 10.1038/s44321-024-00145-8 39358604 PMC11554675

[B68] Mei Q. Zhang Y. Li H. Ma W. Huang W. Wu Z. (2025). Hepatic factor MANF drives hepatocytes reprogramming by detaining cytosolic CK19 in intrahepatic cholangiocarcinoma. Cell Death Differ. 32 (8), 1441–1459. 10.1038/s41418-025-01460-4 39972058 PMC12325741

[B69] Meng Y. Sun P. Ren Y. Li G. Liu X. Xu C. (2025). Azvudine suppresses epithelial-mesenchymal transition in hepatocellular carcinoma by targeting the Notch-HEY signalling pathway. Int. J. Mol. Sci. 26 (11), 5127. 10.3390/ijms26115127 40507937 PMC12154575

[B70] Meurette O. Mehlen P. (2018). Notch signaling in the tumor microenvironment. Cancer Cell 34 (4), 536–548. 10.1016/j.ccell.2018.07.009 30146333

[B71] Miao X. Guo Y. Zeng S. Liu X. Teng X. Li L. (2021). HES5-mediated repression of LIGHT transcription may contribute to apoptosis in hepatocytes. Cell Death Discov. 7 (1), 308. 10.1038/s41420-021-00707-6 34689159 PMC8542050

[B72] Minnis-Lyons S. E. Ferreira-González S. Aleksieva N. Man T. Y. Gadd V. L. Williams M. J. (2021). Notch-IGF1 signaling during liver regeneration drives biliary epithelial cell expansion and inhibits hepatocyte differentiation. Sci. Signal 14 (688), eaay9185. 10.1126/scisignal.aay9185 34158399

[B73] Molina L. M. Zhu J. Li Q. Pradhan-Sundd T. Krutsenko Y. Sayed K. (2021). Compensatory hepatic adaptation accompanies permanent absence of intrahepatic biliary network due to YAP1 loss in liver progenitors. Cell Rep. 36 (1), 109310. 10.1016/j.celrep.2021.109310 34233187 PMC8280534

[B74] Mu R. Chang M. Feng C. Cui Y. Li T. Liu C. (2024). Analysis of the expression of PRDX6 in patients with hepatocellular carcinoma and its effect on the phenotype of hepatocellular carcinoma cells. Curr. Genomics 25 (1), 2–11. 10.2174/0113892029273682240111052317 38544826 PMC10964084

[B75] Nakano Y. Nakao S. Sueoka M. Kasahara D. Tanno Y. Sumiyoshi H. (2022). Two distinct notch signals, Delta-like 4/Notch1 and Jagged-1/Notch2, antagonistically regulate chemical hepatocarcinogenesis in mice. Commun. Biol. 5 (1), 85. 10.1038/s42003-022-03013-8 35064244 PMC8782997

[B76] Pan B. Wang Z. Zhang X. Shen S. Ke X. Qiu J. (2023). Targeted inhibition of RBPJ transcription complex alleviates the exhaustion of CD8(+) T cells in hepatocellular carcinoma. Commun. Biol. 6 (1), 123. 10.1038/s42003-023-04521-x 36717584 PMC9887061

[B77] Peng B. Huang M. Zhang J. Xiang Y. (2025). Organoids in biliary research: insights into developmental signaling and applications in disease modeling. Front. Cell Dev. Biol. 13, 1656019. 10.3389/fcell.2025.1656019 40970096 PMC12440928

[B78] Pu W. Zhu H. Zhang M. Pikiolek M. Ercan C. Li J. (2023). Bipotent transitional liver progenitor cells contribute to liver regeneration. Nat. Genet. 55 (4), 651–664. 10.1038/s41588-023-01335-9 36914834 PMC10101857

[B79] Pu Q. Yu L. Liu X. Yan H. Xie Y. Cai X. (2024). Prognostic value of CD8(+)T cells related genes and exhaustion regulation of Notch signaling pathway in hepatocellular carcinoma. Front. Immunol. 15, 1375864. 10.3389/fimmu.2024.1375864 38650927 PMC11033358

[B80] Qi R. An H. Yu Y. Zhang M. Liu S. Xu H. (2003). Notch1 signaling inhibits growth of human hepatocellular carcinoma through induction of cell cycle arrest and apoptosis. Cancer Res. 63 (23), 8323–8329. 14678992

[B81] Rani J. Dhull S. B. Rose P. K. Kidwai M. K. (2024). Drug-induced liver injury and anti-hepatotoxic effect of herbal compounds: a metabolic mechanism perspective. Phytomedicine 122, 155142. 10.1016/j.phymed.2023.155142 37913641

[B82] Sachan N. Sharma V. Mutsuddi M. Mukherjee A. (2024). Notch signalling: multifaceted role in development and disease. Febs J. 291 (14), 3030–3059. 10.1111/febs.16815 37166442

[B83] Saleh R. O. Alkhafaji A. T. Mohammed J. S. Bansal P. Kaur H. Ahmad I. (2024). LncRNA NEAT1 in the pathogenesis of liver-related diseases. Cell Biochem. Funct. 42 (3), e4006. 10.1002/cbf.4006 38622913

[B84] Schwabe R. F. Tabas I. Pajvani U. B. (2020). Mechanisms of fibrosis development in nonalcoholic steatohepatitis. Gastroenterology 158 (7), 1913–1928. 10.1053/j.gastro.2019.11.311 32044315 PMC7682538

[B85] Shang T. Chen X. Xue H. Wu Y. Lin S. Zhu Y. (2024). The PKHD1 gene inhibits tumor proliferation and invasion in intrahepatic cholangiocarcinoma by activating the Notch pathway. Int. J. Med. Sci. 21 (14), 2655–2663. 10.7150/ijms.95964 39512694 PMC11539381

[B86] Shi J. Han G. Wang J. Han X. Zhao M. Duan X. (2020). Matrine promotes hepatic oval cells differentiation into hepatocytes and alleviates liver injury by suppression of Notch signalling pathway. Life Sci. 261, 118354. 10.1016/j.lfs.2020.118354 32866517

[B87] Shi Q. Jiang S. Zeng Y. Yuan X. Zhang Y. Chu Q. (2024a). A notch signaling pathway-related gene signature: characterizing the immune microenvironment and predicting prognosis in hepatocellular carcinoma. J. Transl. Int. Med. 12 (6), 553–568. 10.1515/jtim-2024-0020 40708676 PMC12288947

[B88] Shi Q. Xue C. Zeng Y. Yuan X. Chu Q. Jiang S. (2024b). Notch signaling pathway in cancer: from mechanistic insights to targeted therapies. Signal Transduct. Target Ther. 9 (1), 128. 10.1038/s41392-024-01828-x 38797752 PMC11128457

[B89] Shu Y. Xu Q. Xu Y. Tao Q. Shao M. Cao X. (2021). Loss of Numb promotes hepatic progenitor expansion and intrahepatic cholangiocarcinoma by enhancing Notch signaling. Cell Death Dis. 12 (11), 966. 10.1038/s41419-021-04263-w 34667161 PMC8526591

[B90] Sparks E. E. Huppert K. A. Brown M. A. Washington M. K. Huppert S. S. (2010). Notch signaling regulates formation of the three-dimensional architecture of intrahepatic bile ducts in mice. Hepatology 51 (4), 1391–1400. 10.1002/hep.23431 20069650 PMC2995854

[B91] Sparling D. P. Yu J. Kim K. Zhu C. Brachs S. Birkenfeld A. L. (2016). Adipocyte-specific blockade of gamma-secretase, but not inhibition of notch activity, reduces adipose insulin sensitivity. Mol. Metab. 5 (2), 113–121. 10.1016/j.molmet.2015.11.006 26909319 PMC4735659

[B92] Sprinzak D. Blacklow S. C. (2021). Biophysics of notch signaling. Annu. Rev. Biophys. 50, 157–189. 10.1146/annurev-biophys-101920-082204 33534608 PMC8105286

[B93] Steinman J. B. Salomao M. A. Pajvani U. B. (2021). Zonation in NASH - a key paradigm for understanding pathophysiology and clinical outcomes. Liver Int. 41 (11), 2534–2546. 10.1111/liv.15025 34328687

[B94] Suarez Rodriguez F. Sanlidag S. Sahlgren C. (2023). Mechanical regulation of the Notch signaling pathway. Curr. Opin. Cell Biol. 85, 102244. 10.1016/j.ceb.2023.102244 37783031

[B95] Sun J. Dong M. Xiang X. Zhang S. Wen D. (2024a). Notch signaling and targeted therapy in non-small cell lung cancer. Cancer Letters 585, 216647. 10.1016/j.canlet.2024.216647 38301911

[B96] Sun L. He M. Li F. Wu D. Zheng P. Zhang C. (2024b). Oxyberberine sensitizes liver cancer cells to sorafenib via inhibiting NOTCH1-USP7-c-Myc pathway. Hepatol. Commun. 8 (4), e0405. 10.1097/hc9.0000000000000405 38573832 PMC10997235

[B97] Sun Y. Weng X. Chen W. Ge J. Ding B. Ru J. (2025). MYBBP1A-mediated IGFBP4 promoter methylation promotes epithelial-mesenchymal transition and metastasis through activation of NOTCH pathway in liver cancer. Int. J. Oncol. 66 (1), 4. 10.3892/ijo.2024.5710 39611481 PMC11637501

[B98] Tao W. Zhu W. Nabi F. Li Z. Liu J. (2023). Penthorum chinense pursh compound flavonoids supplementation alleviates Aflatoxin B1-induced liver injury via modulation of intestinal barrier and gut microbiota in broiler. Ecotoxicol. Environ. Saf. 255, 114805. 10.1016/j.ecoenv.2023.114805 36958264

[B99] Turnpenny P. D. Ellard S. (2012). Alagille syndrome: pathogenesis, diagnosis and management. Eur. J. Hum. Genet. 20 (3), 251–257. 10.1038/ejhg.2011.181 21934706 PMC3283172

[B100] Valizadeh A. Majidinia M. Samadi-Kafil H. Yousefi M. Yousefi B. (2019). The roles of signaling pathways in liver repair and regeneration. J. Cell Physiol. 234 (9), 14966–14974. 10.1002/jcp.28336 30770551

[B101] Valizadeh A. Sayadmanesh A. Asemi Z. Alemi F. Mahmoodpoor A. Yousefi B. (2021). Regulatory roles of the notch signaling pathway in liver repair and regeneration: a novel therapeutic target. Curr. Med. Chem. 28 (41), 8608–8626. 10.2174/0929867328666210419123200 33874861

[B102] Viatour P. Ehmer U. Saddic L. A. Dorrell C. Andersen J. B. Lin C. (2011). Notch signaling inhibits hepatocellular carcinoma following inactivation of the RB pathway. J. Exp. Med. 208 (10), 1963–1976. 10.1084/jem.20110198 21875955 PMC3182062

[B103] Wang F. S. Fan J. G. Zhang Z. Gao B. Wang H. Y. (2014). The global burden of liver disease: the major impact of China. Hepatology 60 (6), 2099–2108. 10.1002/hep.27406 25164003 PMC4867229

[B104] Wang T. Xu C. Zhang Z. Wu H. Li X. Zhang Y. (2022). Cellular heterogeneity and transcriptomic profiles during intrahepatic cholangiocarcinoma initiation and progression. Hepatology 76 (5), 1302–1317. 10.1002/hep.32483 35340039 PMC9790314

[B105] Wu H. Liu S. Wu D. Zhou H. Sui G. Wu G. (2023). Cell division cycle-associated 8 is a prognostic biomarker related to immune invasion in hepatocellular carcinoma. Cancer Med. 12 (8), 10138–10155. 10.1002/cam4.5718 36855818 PMC10166956

[B106] Xi Z. Zhang J. Bao M. (2025). Notch signaling in metabolic diseases: a key regulator and potential target. Biochem. Biophys. Res. Commun. 781, 152492. 10.1016/j.bbrc.2025.152492 40834603

[B107] Xiao X. Zhang Q. (2023). Asiaticoside conveys an antifibrotic effect by inhibiting activation of hepatic stellate cells via the Jagged-1/Notch-1 pathway. J. Nat. Med. 77 (1), 128–136. 10.1007/s11418-022-01653-y 36169781

[B108] Xiao Y. Batmanov K. Hu W. Zhu K. Tom A. Y. Guan D. (2023). Hepatocytes demarcated by EphB2 contribute to the progression of nonalcoholic steatohepatitis. Sci. Transl. Med. 15 (682), eadc9653. 10.1126/scitranslmed.adc9653 36753562 PMC10234568

[B109] Xiao J. Wang F. Yuan Y. Gao J. Xiao L. Yan C. (2025). Epidemiology of liver diseases: global disease burden and forecasted research trends. Sci. China Life Sci. 68 (2), 541–557. 10.1007/s11427-024-2722-2 39425834

[B110] Xin H. Lai Q. Liu Y. Liao N. Wang Y. Liao B. (2024). Integrative radiomics analyses identify universal signature for predicting prognosis and therapeutic vulnerabilities across primary and secondary liver cancers: a multi-cohort study. Pharmacol. Res. 210, 107535. 10.1016/j.phrs.2024.107535 39626849

[B111] Xu H. Wang L. (2021). The role of notch signaling pathway in non-alcoholic fatty liver disease. Front. Mol. Biosci. 8, 792667. 10.3389/fmolb.2021.792667 34901163 PMC8652134

[B112] Xu J. Yun X. Jiang J. Wei Y. Wu Y. Zhang W. (2010). Hepatitis B virus X protein blunts senescence-like growth arrest of human hepatocellular carcinoma by reducing Notch1 cleavage. Hepatology 52 (1), 142–154. 10.1002/hep.23613 20578140

[B113] Xu W. Xu Y. N. Zhang X. Xu Y. Jian X. Chen J. M. (2020). Hepatic stem cell Numb gene is a potential target of Huang Qi decoction against cholestatic liver fibrosis. Sci. Rep. 10 (1), 17486. 10.1038/s41598-020-74324-1 33060633 PMC7566460

[B114] Xu D. Qu X. Tian Y. Jie Z. Xi Z. Xue F. (2022). Macrophage Notch1 inhibits TAK1 function and RIPK3-mediated hepatocyte necroptosis through activation of β-catenin signaling in liver ischemia and reperfusion injury. Cell Commun. Signal 20 (1), 144. 10.1186/s12964-022-00901-8 36114543 PMC9479434

[B115] Xu M. Zheng Y. Chen J. Gao C. Zhu M. Ma A. (2025). CLDN4 palmitoylation promotes hepatic-to-biliary lineage transition and lenvatinib resistance in hepatocellular carcinoma. Cell Rep. Med. 6 (7), 102208. 10.1016/j.xcrm.2025.102208 40592346 PMC12281411

[B116] Yan B. Lu Q. Gao T. Xiao K. Zong Q. Lv H. (2025). CD146 regulates the stemness and chemoresistance of hepatocellular carcinoma via JAG2-NOTCH signaling. Cell Death Dis. 16 (1), 150. 10.1038/s41419-025-07470-x 40032820 PMC11876685

[B117] Yang T. Wu E. Zhu X. Leng Y. Ye S. Dong R. (2022). TKF, a mexicanolide-type limonoid derivative, suppressed hepatic stellate cells activation and liver fibrosis through inhibition of the YAP/Notch3 pathway. Phytomedicine 107, 154466. 10.1016/j.phymed.2022.154466 36182796

[B118] Yang T. Qu X. Zhao J. Wang X. Wang Q. Dai J. (2023). Macrophage PTEN controls STING-induced inflammation and necroptosis through NICD/NRF2 signaling in APAP-induced liver injury. Cell Commun. Signal 21 (1), 160. 10.1186/s12964-023-01175-4 37370115 PMC10294406

[B119] Yasen A. Feng J. Xie X. M. Li K. Cai Y. H. Liao Z. H. (2023). Exosomes derived from TGF-β1-pretreated mesenchymal stem cells alleviate biliary ischemia-reperfusion injury through Jagged1/Notch1/SOX9 pathway. Int. Immunopharmacol. 119, 110253. 10.1016/j.intimp.2023.110253 37156030

[B120] Yu J. Zhu C. Wang X. Kim K. Bartolome A. Dongiovanni P. (2021). Hepatocyte TLR4 triggers inter-hepatocyte Jagged1/Notch signaling to determine NASH-induced fibrosis. Sci. Transl. Med. 13 (599), eabe1692. 10.1126/scitranslmed.abe1692 34162749 PMC8792974

[B121] Yu M. Zhou M. Li J. Zong R. Yan Y. Kong L. (2022). Notch-activated mesenchymal stromal/stem cells enhance the protective effect against acetaminophen-induced acute liver injury by activating AMPK/SIRT1 pathway. Stem Cell Res. Ther. 13 (1), 318. 10.1186/s13287-022-02999-6 35842731 PMC9288678

[B122] Yue Z. Jiang Z. Ruan B. Duan J. Song P. Liu J. (2021). Disruption of myofibroblastic notch signaling attenuates liver fibrosis by modulating fibrosis progression and regression. Int. J. Biol. Sci. 17 (9), 2135–2146. 10.7150/ijbs.60056 34239344 PMC8241719

[B123] Zhan P. Lu Y. Lu J. Cheng Y. Luo C. Yang F. (2024). The activation of the Notch signaling pathway by UBE2C promotes the proliferation and metastasis of hepatocellular carcinoma. Sci. Rep. 14 (1), 22859. 10.1038/s41598-024-72714-3 39353974 PMC11445553

[B124] Zhang P. Yue K. Liu X. Yan X. Yang Z. Duan J. (2020). Endothelial Notch activation promotes neutrophil transmigration via downregulating endomucin to aggravate hepatic ischemia/reperfusion injury. Sci. China Life Sci. 63 (3), 375–387. 10.1007/s11427-019-1596-4 32048161

[B125] Zhang X. Xu Z. Chen Q. Zhou Z. (2024a). Notch signaling regulates pulmonary fibrosis. Front. Cell Dev. Biol. 12, 1450038. 10.3389/fcell.2024.1450038 39450276 PMC11499121

[B126] Zhang Y. Qu J. Luo R. Jia K. Fan G. Li F. (2024b). Radix rehmanniae praeparata extracts ameliorate hepatic ischemia-reperfusion injury by reversing LRP1-NOTCH1-C/EBPβ axis-mediated senescence fate of LSECs. Phytomedicine 133, 155923. 10.1016/j.phymed.2024.155923 39094438

[B127] Zhang L. Niknejad N. Li J. Peng L. Jain S. Steiner D. (2025a). Antisense oligonucleotide-mediated upregulation of Jag1 ameliorates liver disease phenotypes in a mouse model of Alagille syndrome. Mol. Ther. Nucleic Acids 36 (4), 102694. 10.1016/j.omtn.2025.102694 40980686 PMC12446204

[B128] Zhang X. Li S. Hao L. Jia F. Yu F. Hu X. (2025b). Influencing factors and mechanism of hepatocyte regeneration. J. Transl. Med. 23 (1), 493. 10.1186/s12967-025-06278-9 40307789 PMC12042435

[B129] Zhao C. Matalonga J. Lancman J. J. Liu L. Xiao C. Kumar S. (2022). Regenerative failure of intrahepatic biliary cells in Alagille syndrome rescued by elevated Jagged/Notch/Sox9 signaling. Proc. Natl. Acad. Sci. U. S. A. 119 (50), e2201097119. 10.1073/pnas.2201097119 36469766 PMC9897440

[B130] Zhong Y. J. Luo X. M. Liu F. He Z. Q. Yang S. Q. Ma W. J. (2024). Integrative analyses of bulk and single-cell transcriptomics reveals the infiltration and crosstalk of cancer-associated fibroblasts as a novel predictor for prognosis and microenvironment remodeling in intrahepatic cholangiocarcinoma. J. Transl. Med. 22 (1), 422. 10.1186/s12967-024-05238-z 38702814 PMC11071156

[B131] Zhou B. Lin W. Long Y. Yang Y. Zhang H. Wu K. (2022). Notch signaling pathway: architecture, disease, and therapeutics. Signal Transduct. Target Ther. 7 (1), 95. 10.1038/s41392-022-00934-y 35332121 PMC8948217

[B132] Zhou C. Weng J. Liu C. Liu S. Hu Z. Xie X. (2023a). Disruption of SLFN11 deficiency-induced CCL2 signaling and macrophage M2 polarization potentiates Anti-PD-1 therapy efficacy in hepatocellular carcinoma. Gastroenterology 164 (7), 1261–1278. 10.1053/j.gastro.2023.02.005 36863689

[B133] Zhou Q. Li B. Li J. (2023b). DLL4-Notch signalling in acute-on-chronic liver failure: state of the art and perspectives. Life Sci. 317, 121438. 10.1016/j.lfs.2023.121438 36709913

[B134] Zhu C. Kim K. Wang X. Bartolome A. Salomao M. Dongiovanni P. (2018). Hepatocyte Notch activation induces liver fibrosis in nonalcoholic steatohepatitis. Sci. Transl. Med. 10 (468), eaat0344. 10.1126/scitranslmed.aat0344 30463916 PMC6822168

[B135] Zhu C. Ma J. Zhu K. Yu L. Zheng B. Rao D. (2023). Spatial immunophenotypes predict clinical outcome in intrahepatic cholangiocarcinoma. JHEP Rep. 5 (8), 100762. 10.1016/j.jhepr.2023.100762 37360908 PMC10285646

